# Supplementary material for: Sulforaphene suppressed cell proliferation and promoted apoptosis of COV362 cells in endometrioid ovarian cancer
Source: PeerJ. 2023 Nov 21;11:e16308. doi: 10.7717/peerj.16308 (PMC10668859; doi:10.7717/peerj.16308)
Supplement: Supplemental Information 2 [file peerj-11-16308-s002.docx]

| **ITEM TO CHECK** | **IMPORTANCE** | **CHECKLIST** |
| --- | --- | --- |
| **EXPERIMENTAL DESIGN** |  |  |
| Deﬁnition of experimental and control groups | **E** | Line 130 |
| Number within each group | **E** | Line 130 |
| Assay carried out by core lab or investigator's lab? | D | NA |
| Acknowledgement of authors'contributions | D | Line 346-347 |
| **SAMPLE** |  |  |
| Description | **E** | Line 130 |
| Volume/mass of sample processed | D | Line 130 |
| Microdissection or macrodissection | **E** | NA |
| Processing procedure | **E** | NA |
| If frozen - how and how quickly? | **E** | NA |
| If ﬁxed - with what, how quickly? | **E** | NA |
| Sample storage conditions and duration (especially for FFPE samples) | **E** | NA |
| **NUCLEIC ACID EXTRACTION** |  |  |
| Procedure and/or instrumentation | **E** | Line 130 |
| Name of kit and details of any modiﬁcations | **E** | Line 130 |
| Source of additional reagents used | D | Line 130 |
| Details of DNase or RNAse treatment | **E** | Line 130 |
| Contamination assessment (DNA or RNA) | **E** | NA |
| Nucleic acid quantiﬁcation | **E** | Line 131-132 |
| Instrument and method | **E** | Line 131-132 |
| Purity (A260/A280) | D | Line 131-132 |
| Yield | D | Line 131-132 |
| RNA integrity method/instrument | **E** | NA |
| RIN/RQI or Cq of 3' and 5' transcripts | **E** | NA |
| Electrophoresis traces | D | NA |
| Inhibition testing (Cq dilutions, spike or other) | **E** | NA |
| **REVERSE TRANSCRIPTION** |  |  |
| Complete reaction conditions | **E** | Line 132-133 |
| Amount of RNA and reaction volume | **E** | Line 132-133 |
| Priming oligonucleotide (if using GSP) and concentration | **E** | Line 132-133 |
| Reverse transcriptase and concentration | **E** | Line 132-133 |
| Temperature and time | **E** | Line 132-133 |
| Manufacturer of reagents and catalogue numbers | D | NA |
| Cqs with and without RT | D* | NA |
| Storage conditions of cDNA | D | NA |
| **qPCR TARGET INFORMATION** |  |  |
| If multiplex, efﬁciency and LOD of each assay. | **E** | NA |
| Sequence accession number | **E** | NA |
| Location of amplicon | D | NA |
| Amplicon length | **E** | NA |
| *In silico* speciﬁcity screen (BLAST, etc)  Pseudogenes, retropseudogenes or other homologs?  Sequence alignment | **E** | NA |
|  | D | NA |
|  | D | NA |
| Secondary structure analysis of amplicon | D | NA |
| Location of each primer by exon or intron (if applicable) | **E** | NA |
| What splice variants are targeted? | **E** | NA |
| **qPCR OLIGONUCLEOTIDES** |  |  |
| Primer sequences | **E** | Line 137-152 |
| RTPrimerDB Identiﬁcation Number | D | NA |
| Probe sequences | D** | NA |
| Location and identity of any modiﬁcations | **E** | NA |
| Manufacturer of oligonucleotides | D | NA |
| Puriﬁcation method | D | NA |
| **qPCR PROTOCOL** |  |  |
| Complete reaction conditions | **E** | Line 130-134 |
| Reaction volume and amount of cDNA/DNA | **E** | Line 130-134 |
| Primer, (probe), Mg++ and dNTP concentrations | **E** | Line 130-134 |
| Polymerase identity and concentration | **E** | Line 130-134 |
| Buffer/kit identity and manufacturer | **E** | Line 130-134 |
| Exact chemical constitution of the buffer | D | NA |
| Additives (SYBR Green I, DMSO, etc.) | **E** | Line 133-134 |
| Manufacturer of plates/tubes and catalog number | D | NA |
| Complete thermocycling parameters | **E** | Line 134-136 |
| Reaction setup (manual/robotic) | D | NA |
| Manufacturer of qPCR instrument | **E** | Line 134 |
| **qPCR VALIDATION** |  |  |
| Evidence of optimisation (from gradients) | D | NA |
| Speciﬁcity (gel, sequence, melt, or digest) | **E** | NA |
| For SYBR Green I, Cq of the NTC | **E** | NA |
| Standard curves with slope and y-intercept | **E** | NA |
| PCR efﬁciency calculated from slope | **E** | NA |
| Conﬁdence interval for PCR efﬁciency or standard error | D | NA |
| r2 of standard curve | **E** | NA |
| Linear dynamic range | **E** | NA |
| Cq variation at lower limit | **E** | NA |
| Conﬁdence intervals throughout range | D | NA |
| Evidence for limit of detection | **E** | NA |
| If multiplex, efﬁciency and LOD of each assay. | **E** | NA |
| **DATA ANALYSIS** |  |  |
| qPCR analysis program (source, version) | **E** | Line 153-155 |
| Cq method determination | **E** | Line 153-155 |
| Outlier identiﬁcation and disposition | **E** | Line 153-155 |
| Results of NTCs | **E** | Line 153-155 |
| Justiﬁcation of number and choice of reference genes | **E** | Line 153-155 |
| Description of normalisation method | **E** | Line 153-155 |
| Number and concordance of biological replicates | D | NA |
| Number and stage (RT or qPCR) of technical replicates | **E** | Line 153-155 |
| Repeatability (intra-assay variation) | E | NA |
| Reproducibility (inter-assay variation, %CV) | D | NA |
| Power analysis | D | NA |
| Statistical methods for result signiﬁcance | **E** | Line 153-155 |
| Software (source, version) | E | Line 153-155 |
| Cq or raw data submission using RDML | **D** | NA |

**Table 1.** MIQE checklist for authors, reviewers and editors. All essential information (E) must be submitted with the manuscript. Desirable

information (D) should be submitted if available. If using primers obtained from RTPrimerDB, information on qPCR target, oligonucleotides,

protocols and validation is available from that source.

*: Assessing the absence of DNA using a no RT assay is essential when ﬁrst extracting RNA. Once the sample has been validated as

RDNA-free, inclusion of a no-RT control is desirable, but no longer essential.

**: Disclosure of the probe sequence is highly desirable and strongly encouraged. However, since not all commercial pre-designed assay

vendors provide this information, it cannot be an essential requirement. Use of such assays is advised against.
