# Supplementary material for: Sulforaphene suppressed cell proliferation and promoted apoptosis of COV362 cells in endometrioid ovarian cancer
Source: PeerJ. 2023 Nov 21;11:e16308. doi: 10.7717/peerj.16308 (PMC10668859; doi:10.7717/peerj.16308)
Supplement: Supplemental Information 4 [file peerj-11-16308-s004.docx]

**Original uncropped images of western blots used for the Figure 2b.**

**METTL3**


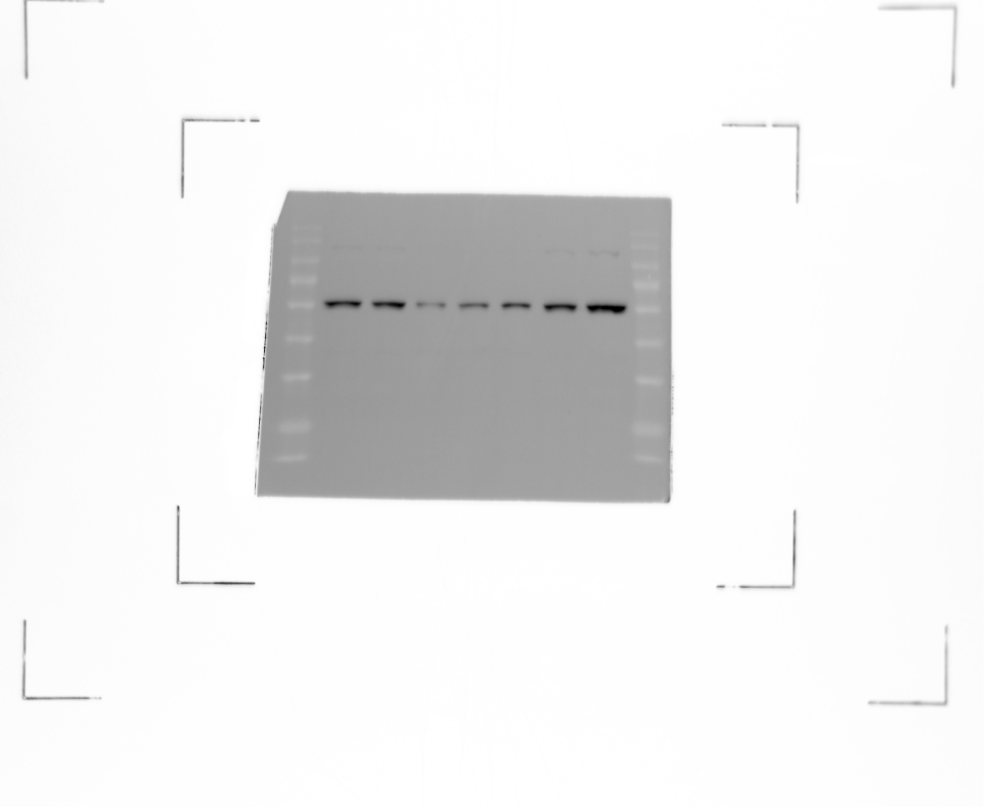


-180 kDa

-63 kDa

-35 kDa

-25 kDa

-17 kDa

-75 kDa

-48 kDa

-135 kDa

-100 kDa

si-METTL3#3

si-NC

Control

si-METTL3#1

si-METTL3#2

pcDNA3.1

pcDNA3.1-METTL3


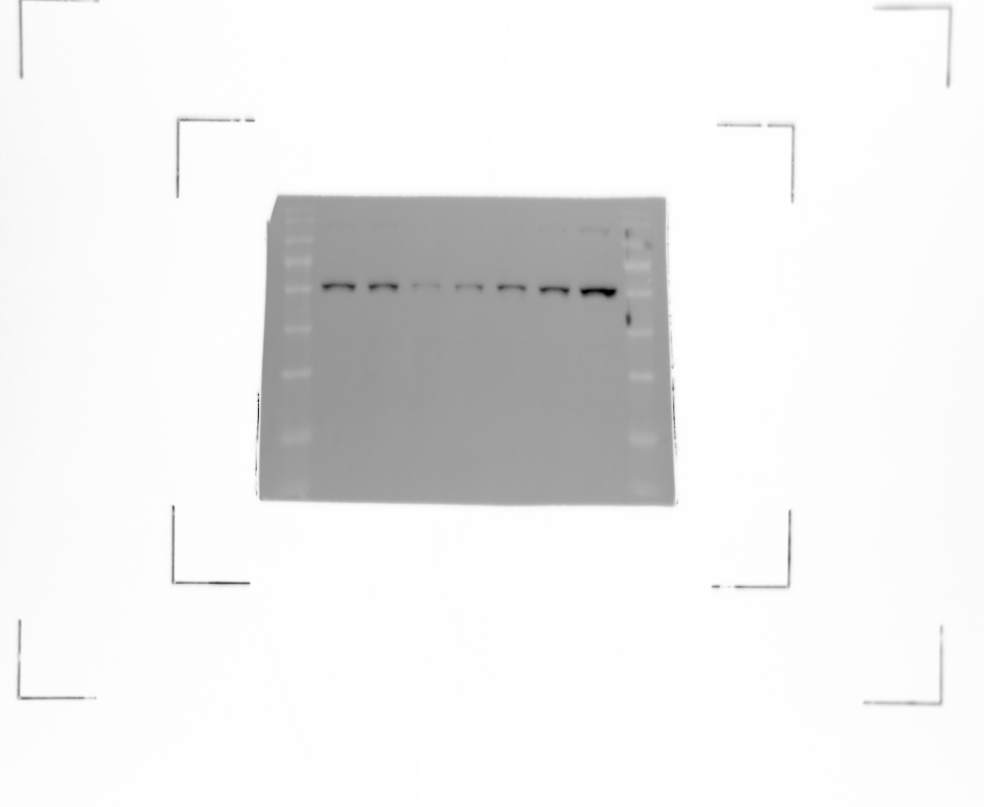


si-METTL3#3

si-NC

Control

si-METTL3#1

si-METTL3#2

pcDNA3.1

pcDNA3.1-METTL3


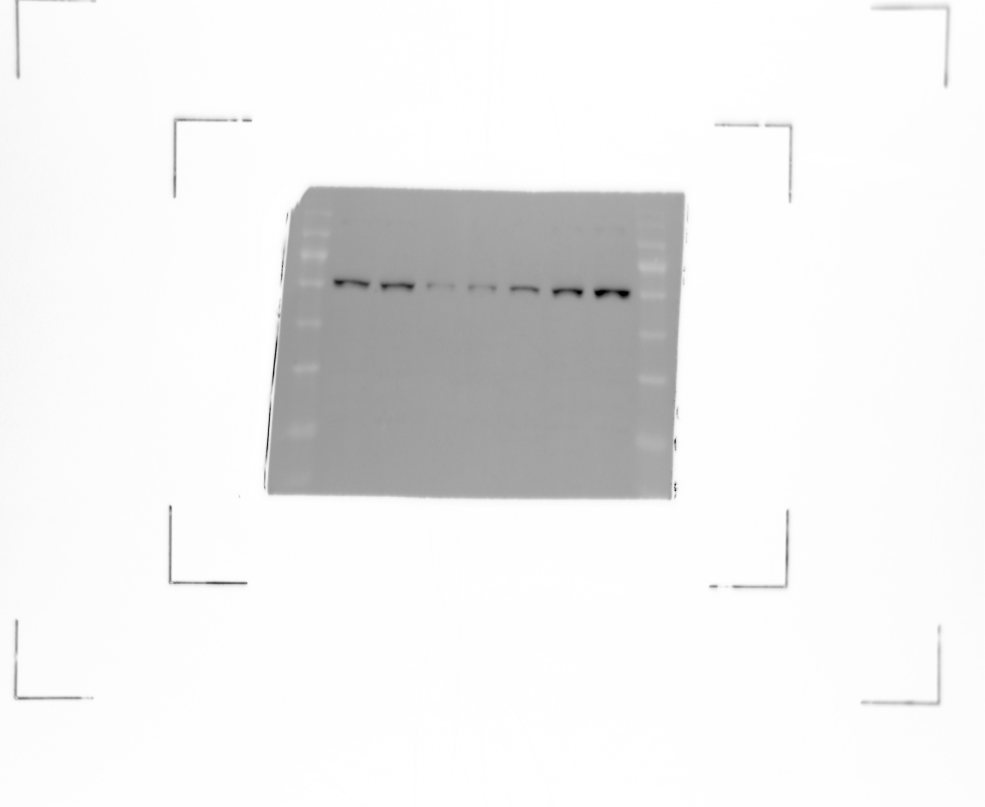


si-METTL3#3

si-NC

Control

si-METTL3#1

si-METTL3#2

pcDNA3.1

pcDNA3.1-METTL3


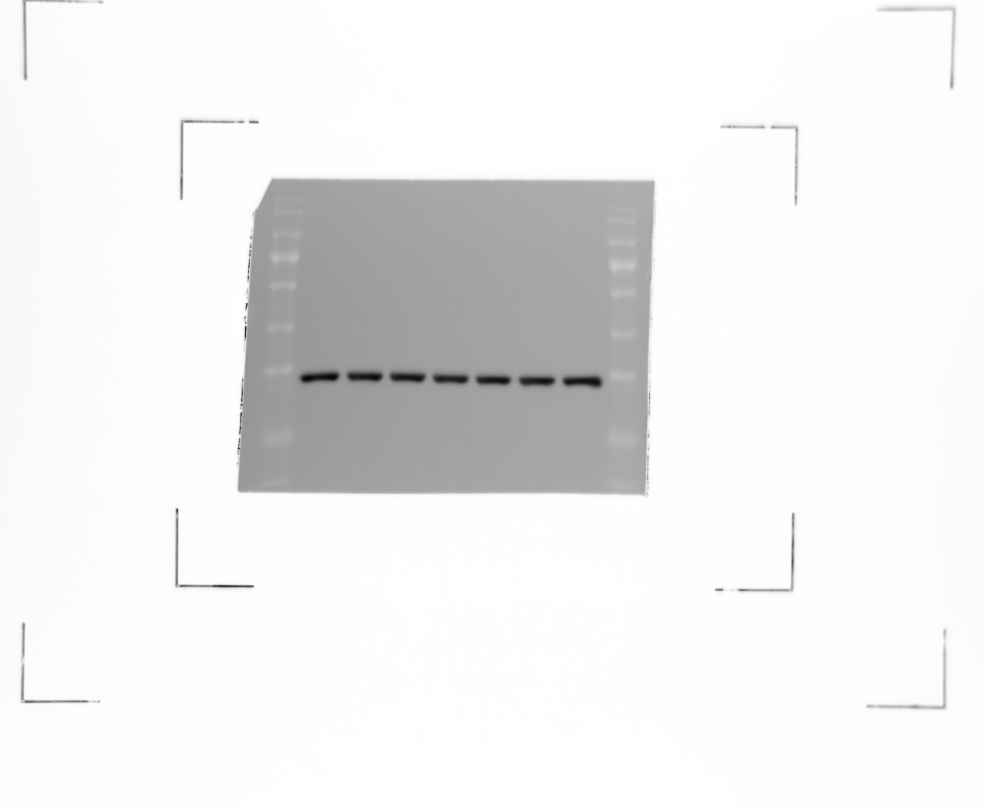


**GAPDH**

si-METTL3#3

si-NC

Control

si-METTL3#1

si-METTL3#2

pcDNA3.1

pcDNA3.1-METTL3

-180 kDa

-63 kDa

-35 kDa

-25 kDa

-17 kDa

-75 kDa

-48 kDa

-135 kDa

-100 kDa


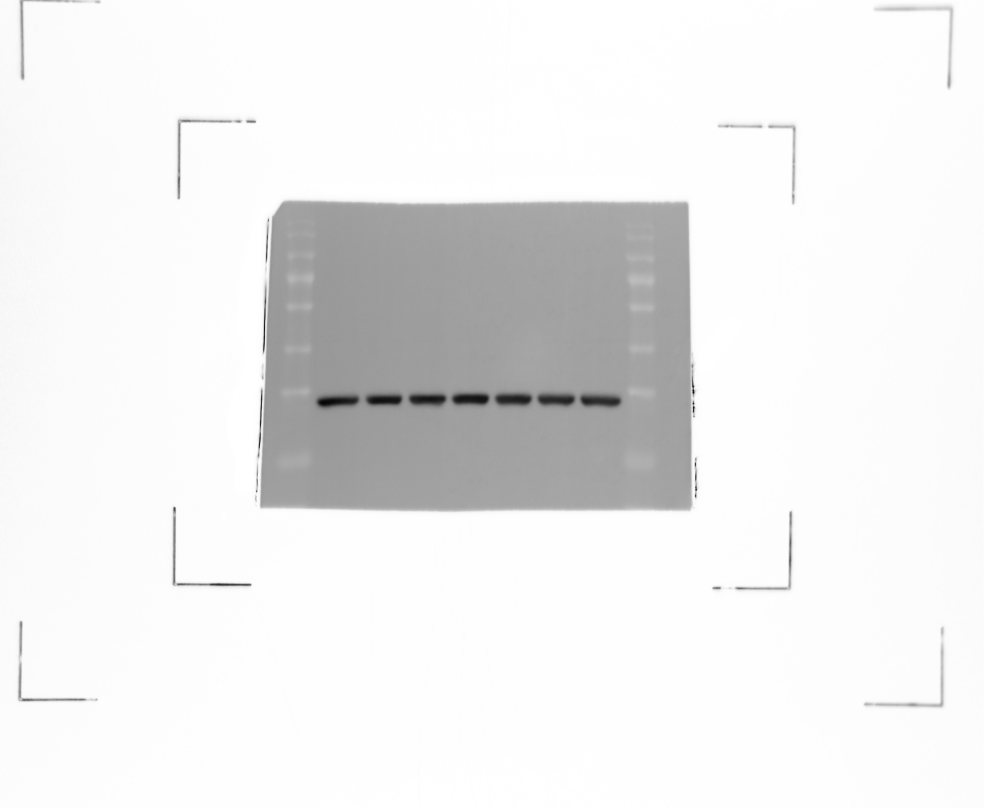


si-METTL3#3

si-NC

Control

si-METTL3#1

si-METTL3#2

pcDNA3.1

pcDNA3.1-METTL3


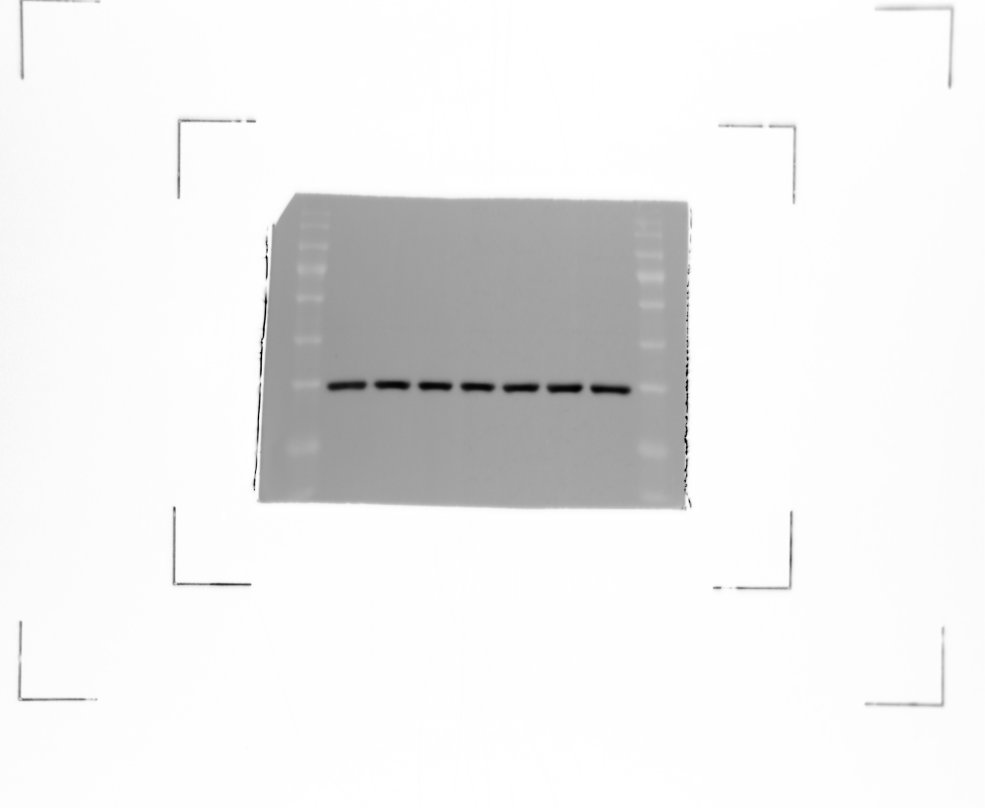


si-METTL3#3

si-NC

Control

si-METTL3#1

si-METTL3#2

pcDNA3.1

pcDNA3.1-METTL3
