# Supplementary material for: Sulforaphene suppressed cell proliferation and promoted apoptosis of COV362 cells in endometrioid ovarian cancer
Source: PeerJ. 2023 Nov 21;11:e16308. doi: 10.7717/peerj.16308 (PMC10668859; doi:10.7717/peerj.16308)
Supplement: Supplemental Information 5 [file peerj-11-16308-s005.docx]

**Original uncropped images of western blots used for the Figure 3b.**

**
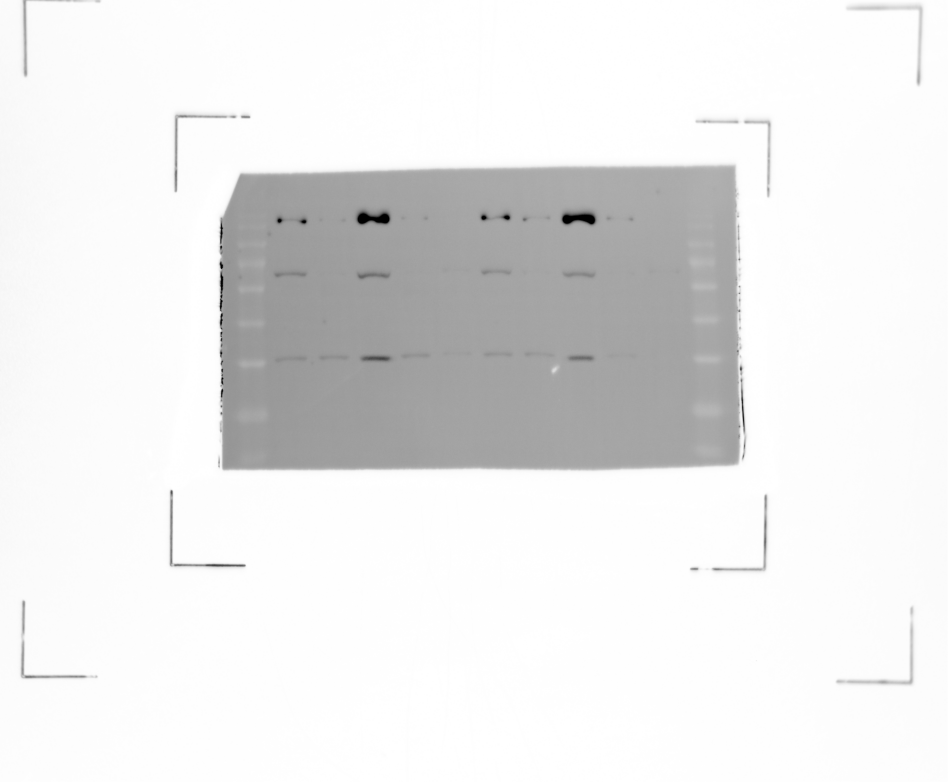
FAS**


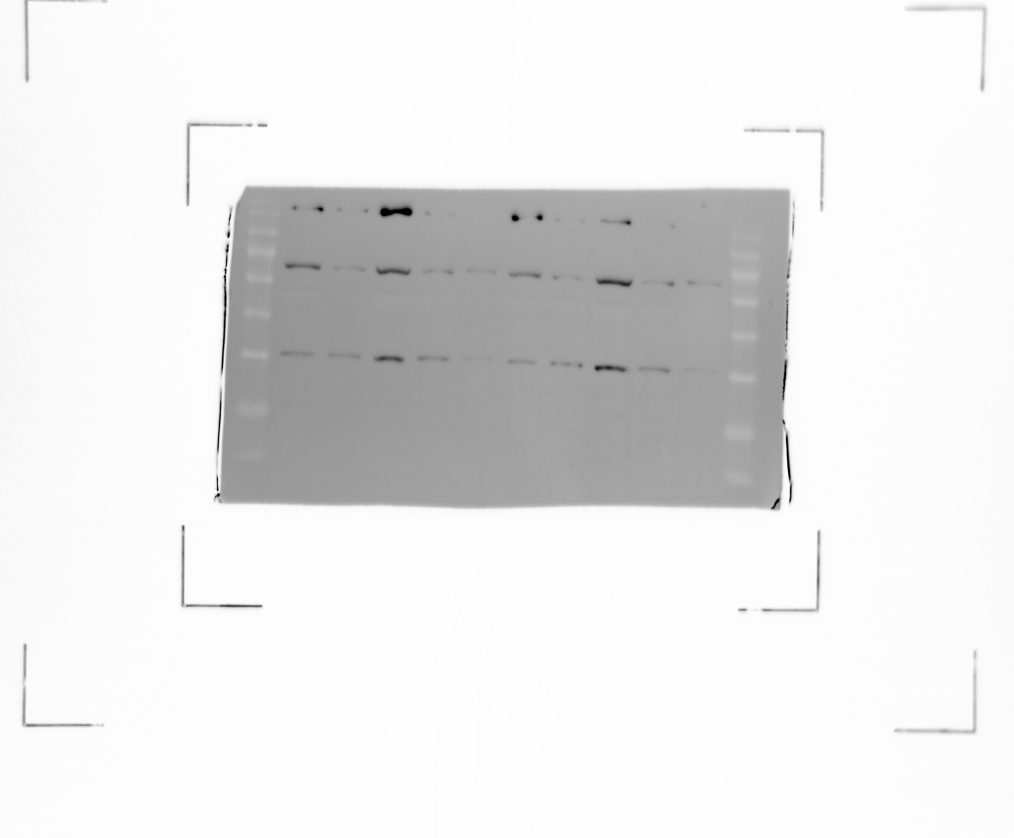


si-NC

Control

si-METTL3#1

pcDNA3.1

pcDNA3.1-METTL3

si-NC

Control

si-METTL3#1

pcDNA3.1

pcDNA3.1-METTL3

si-NC

Control

si-METTL3#1

pcDNA3.1

pcDNA3.1-METTL3

si-NC

Control

si-METTL3#1

pcDNA3.1

pcDNA3.1-METTL3

**FADD**


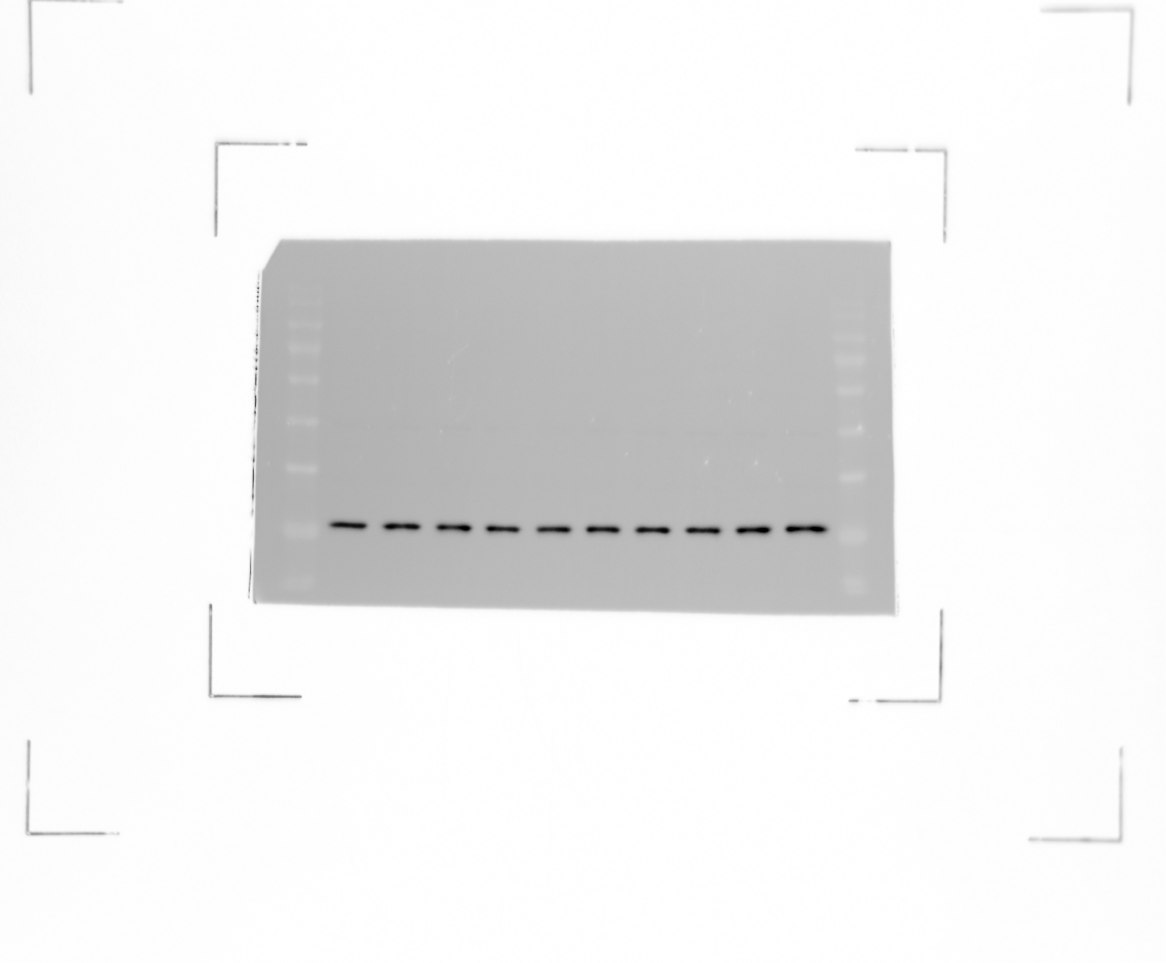

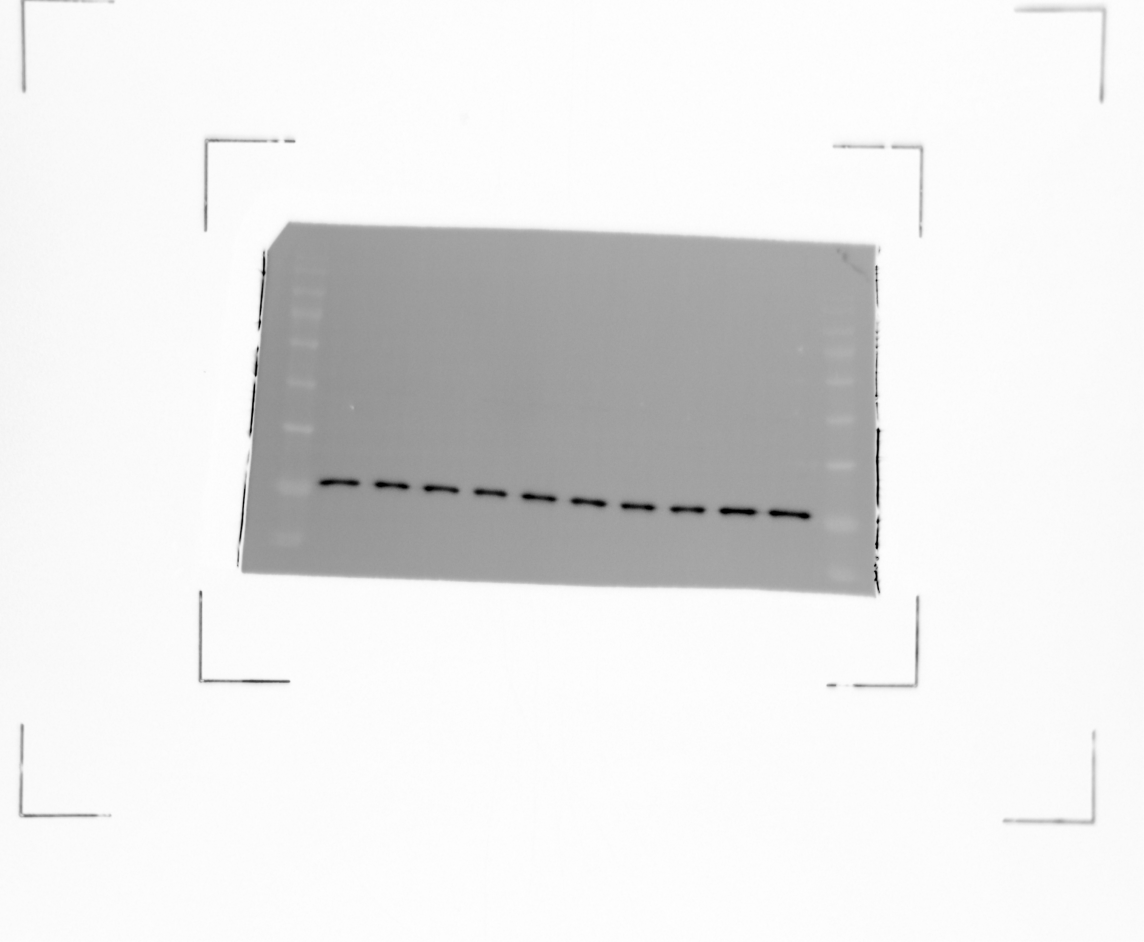


si-NC

Control

si-METTL3#1

pcDNA3.1

pcDNA3.1-METTL3

si-NC

Control

si-METTL3#1

pcDNA3.1

pcDNA3.1-METTL3

si-NC

Control

si-METTL3#1

pcDNA3.1

pcDNA3.1-METTL3

si-NC

Control

si-METTL3#1

pcDNA3.1

pcDNA3.1-METTL3

**p-FADD**


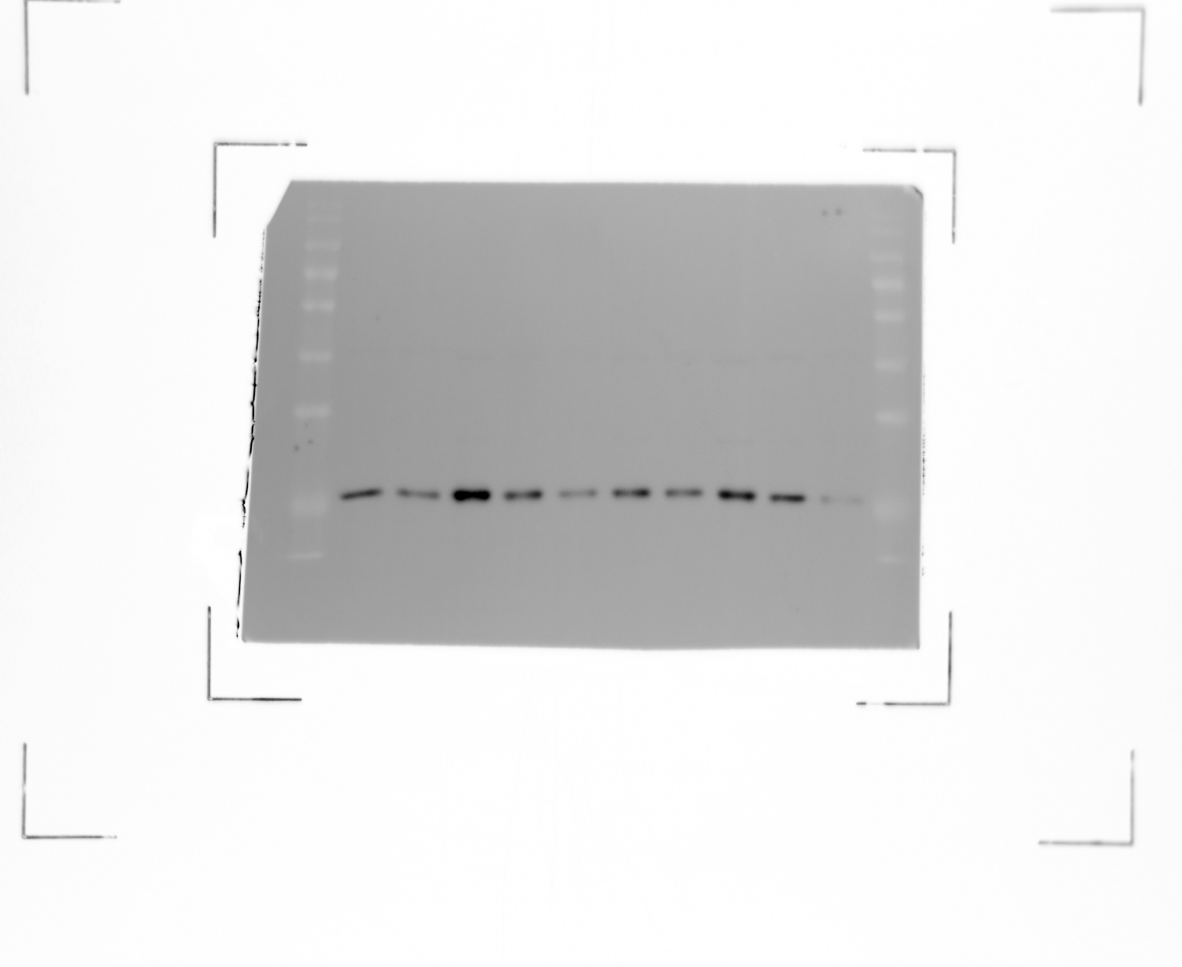

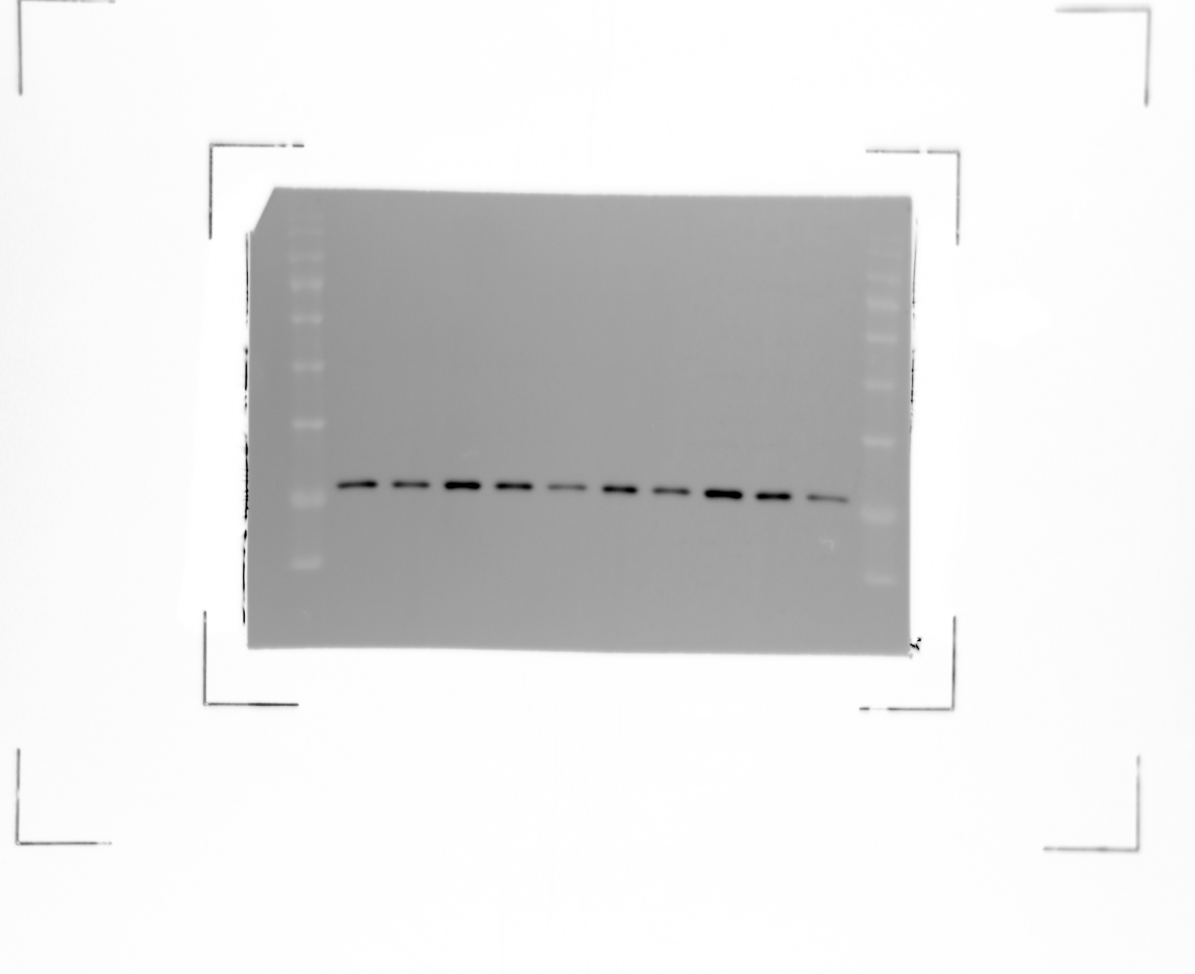


si-NC

Control

si-METTL3#1

pcDNA3.1

pcDNA3.1-METTL3

si-NC

Control

si-METTL3#1

pcDNA3.1

pcDNA3.1-METTL3

si-NC

Control

si-METTL3#1

pcDNA3.1

pcDNA3.1-METTL3

si-NC

Control

si-METTL3#1

pcDNA3.1

pcDNA3.1-METTL3


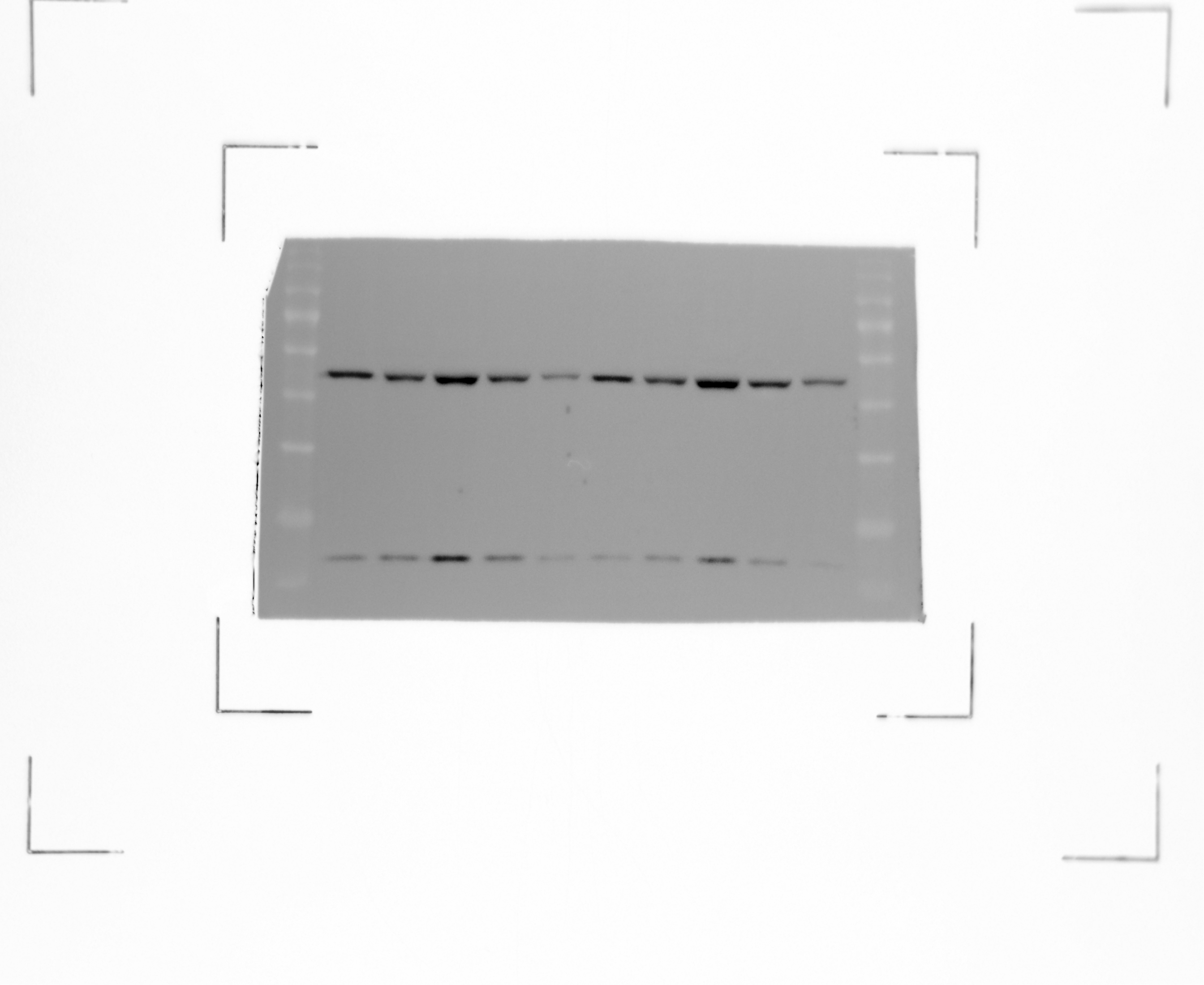


**Caspase-8**


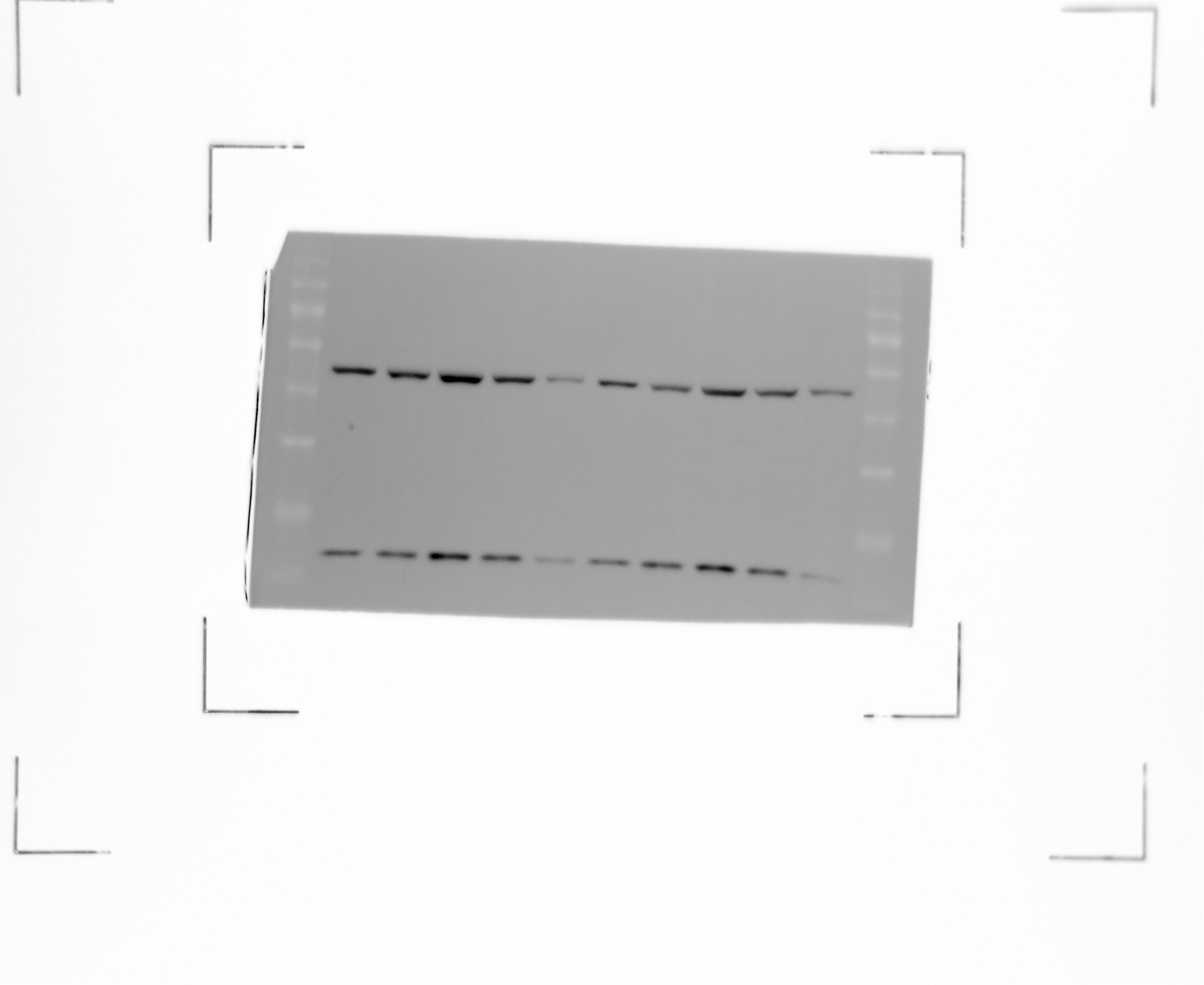


si-NC

Control

si-METTL3#1

pcDNA3.1

pcDNA3.1-METTL3

si-NC

Control

si-METTL3#1

pcDNA3.1

pcDNA3.1-METTL3

si-NC

Control

si-METTL3#1

pcDNA3.1

pcDNA3.1-METTL3

si-NC

Control

si-METTL3#1

pcDNA3.1

pcDNA3.1-METTL3

**Bcl-2**


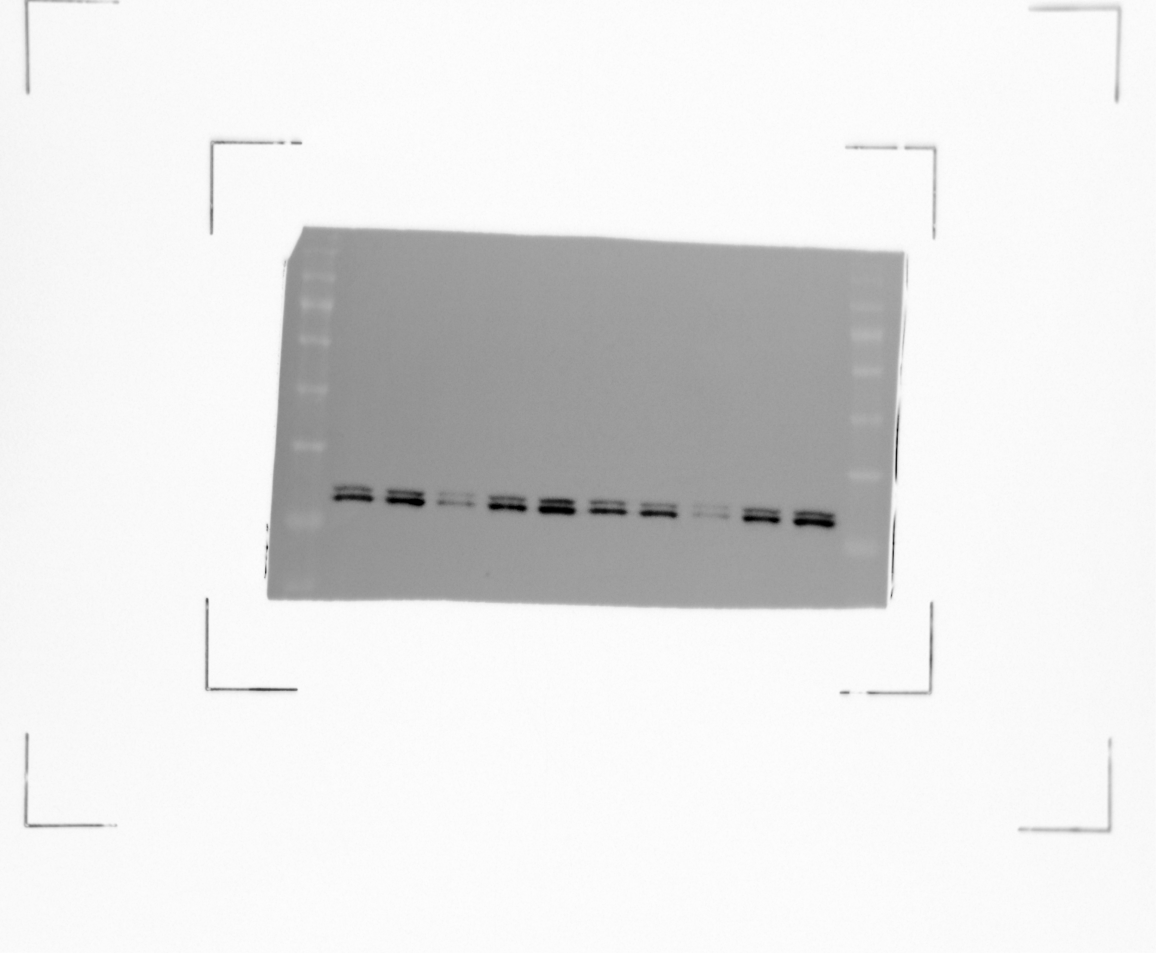

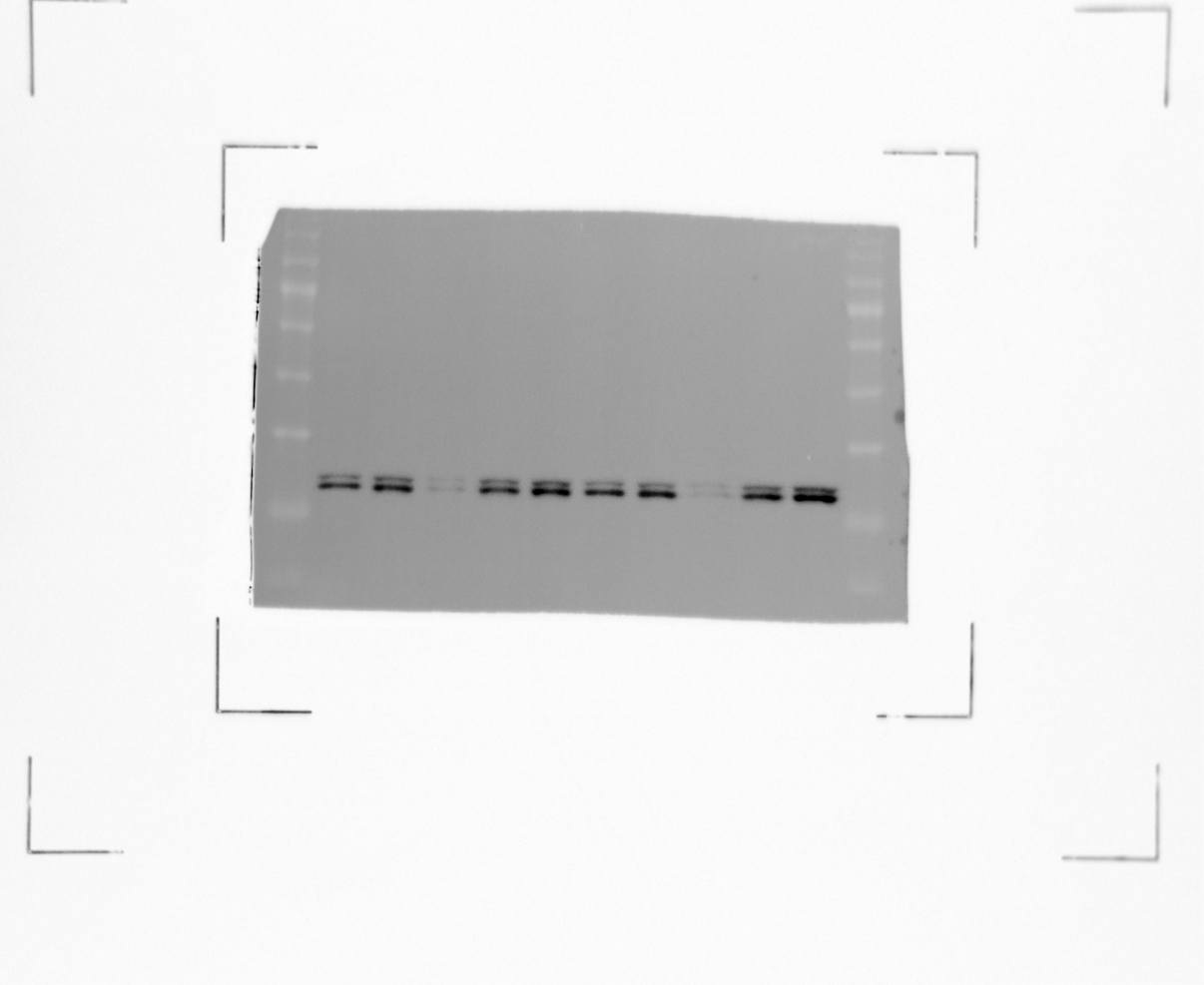


si-NC

Control

si-METTL3#1

pcDNA3.1

pcDNA3.1-METTL3

si-NC

Control

si-METTL3#1

pcDNA3.1

pcDNA3.1-METTL3

si-NC

Control

si-METTL3#1

pcDNA3.1

pcDNA3.1-METTL3

si-NC

Control

si-METTL3#1

pcDNA3.1

pcDNA3.1-METTL3

**
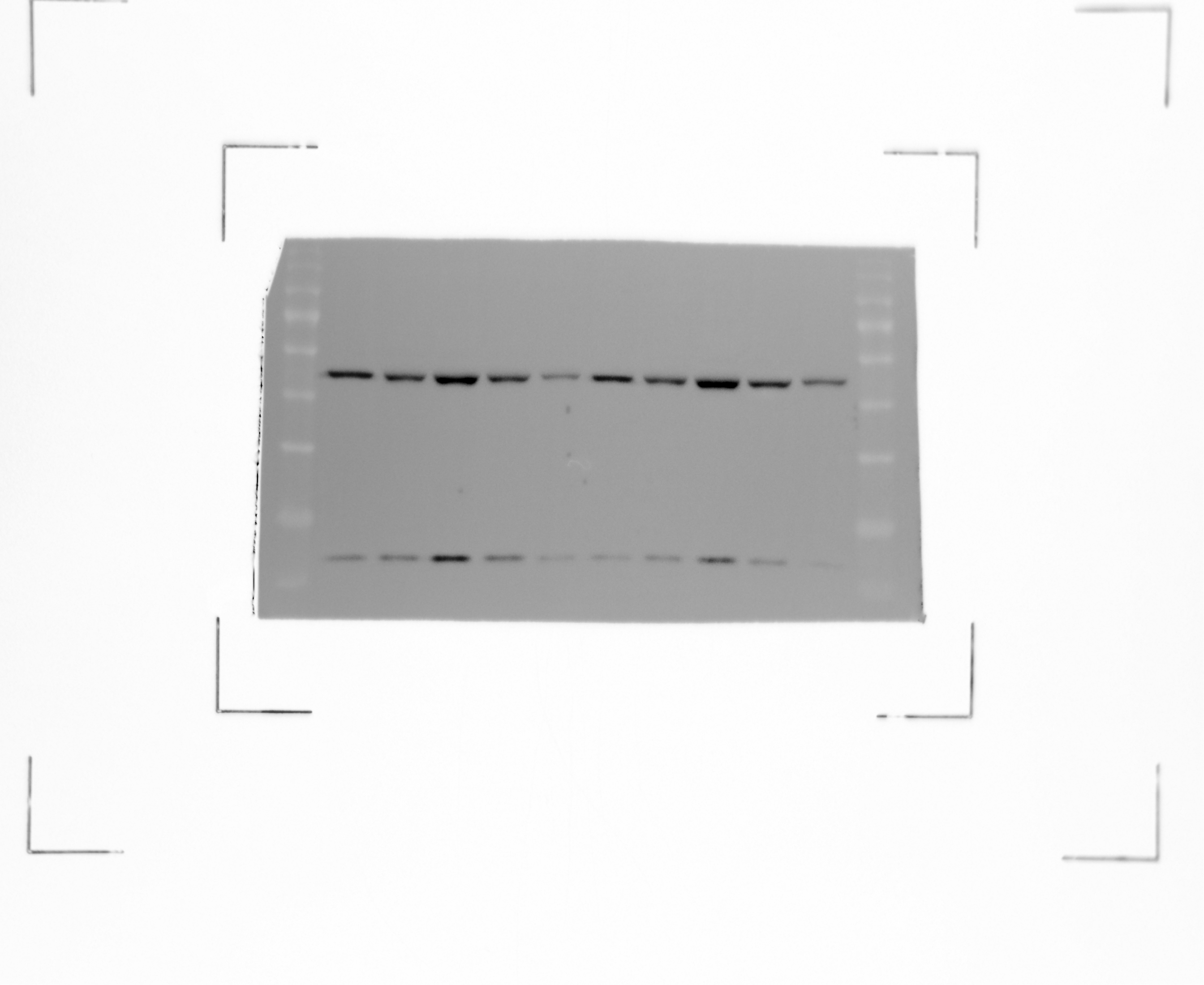
Bax**


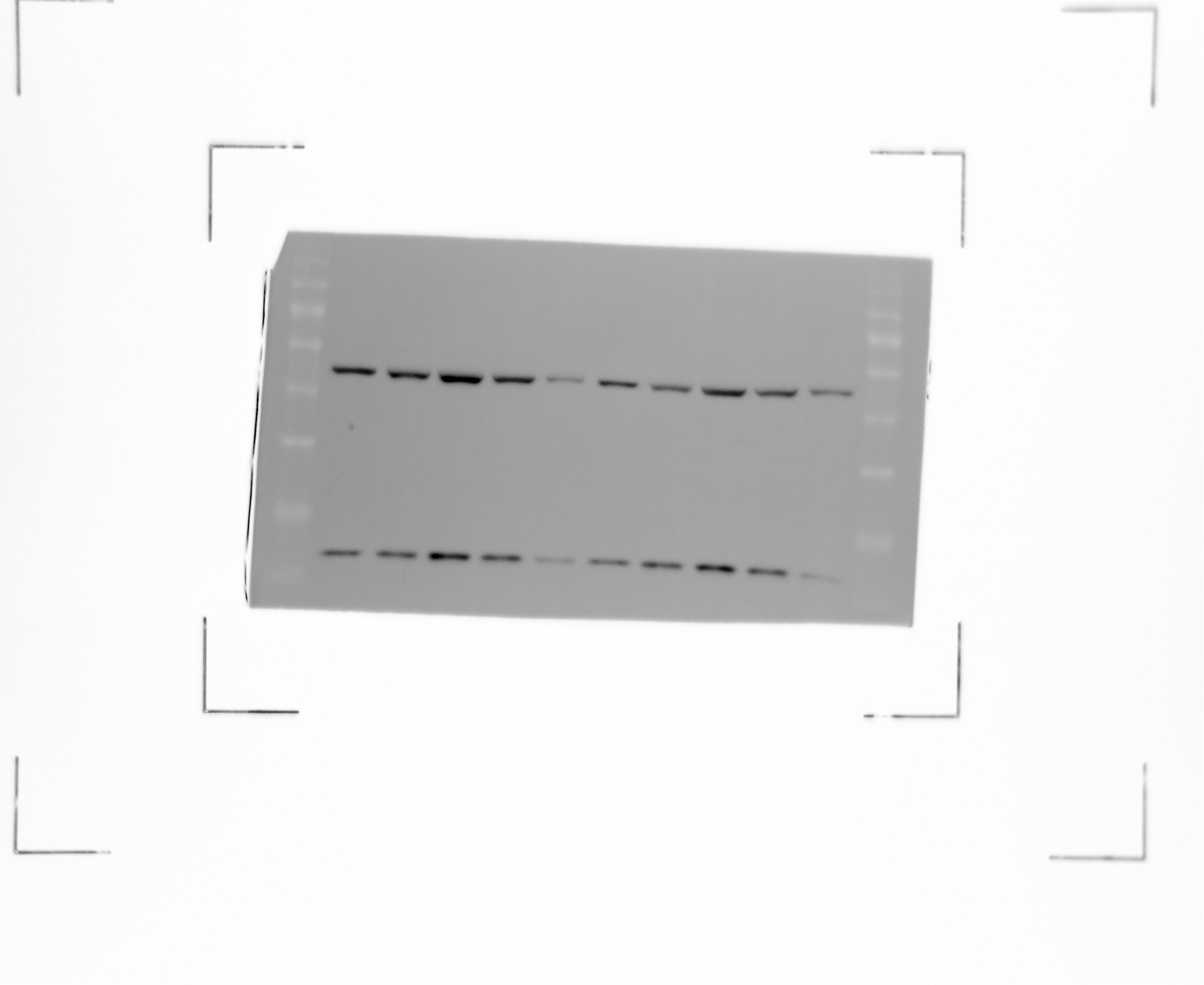


si-NC

Control

si-METTL3#1

pcDNA3.1

pcDNA3.1-METTL3

si-NC

Control

si-METTL3#1

pcDNA3.1

pcDNA3.1-METTL3

si-NC

Control

si-METTL3#1

pcDNA3.1

pcDNA3.1-METTL3

si-NC

Control

si-METTL3#1

pcDNA3.1

pcDNA3.1-METTL3

**GAPDH**


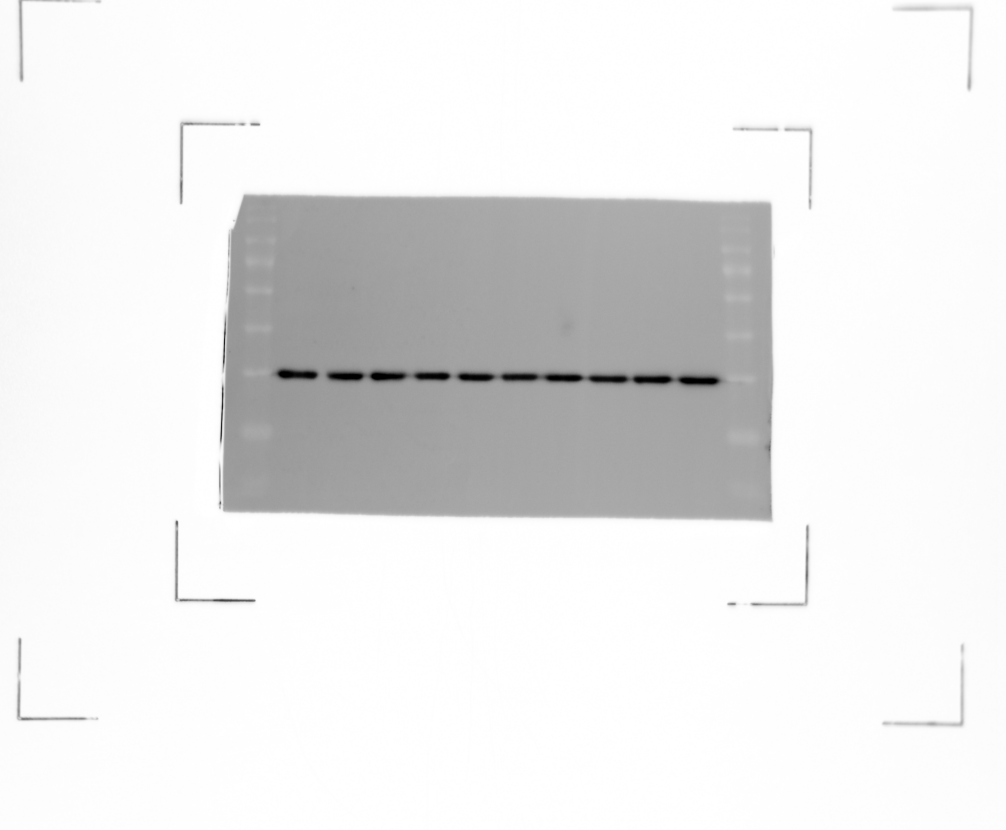

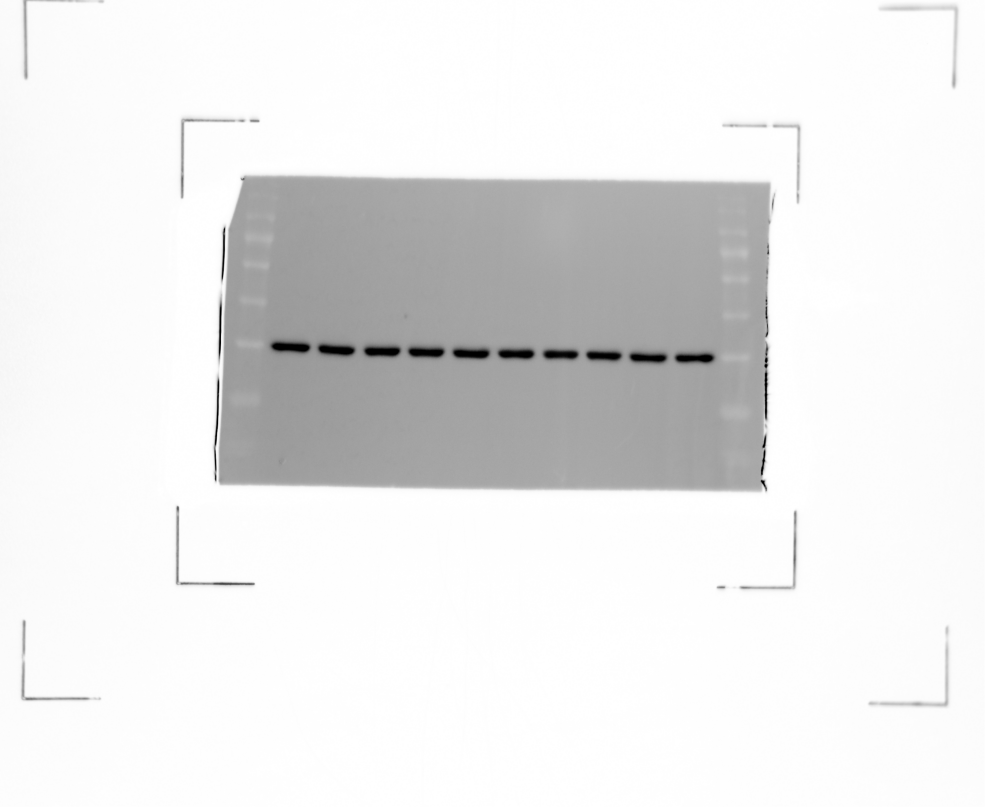


si-NC

Control

si-METTL3#1

pcDNA3.1

pcDNA3.1-METTL3

si-NC

Control

si-METTL3#1

pcDNA3.1

pcDNA3.1-METTL3

si-NC

Control

si-METTL3#1

pcDNA3.1

pcDNA3.1-METTL3

si-NC

Control

si-METTL3#1

pcDNA3.1

pcDNA3.1-METTL3

Cleaved caspase-8

`
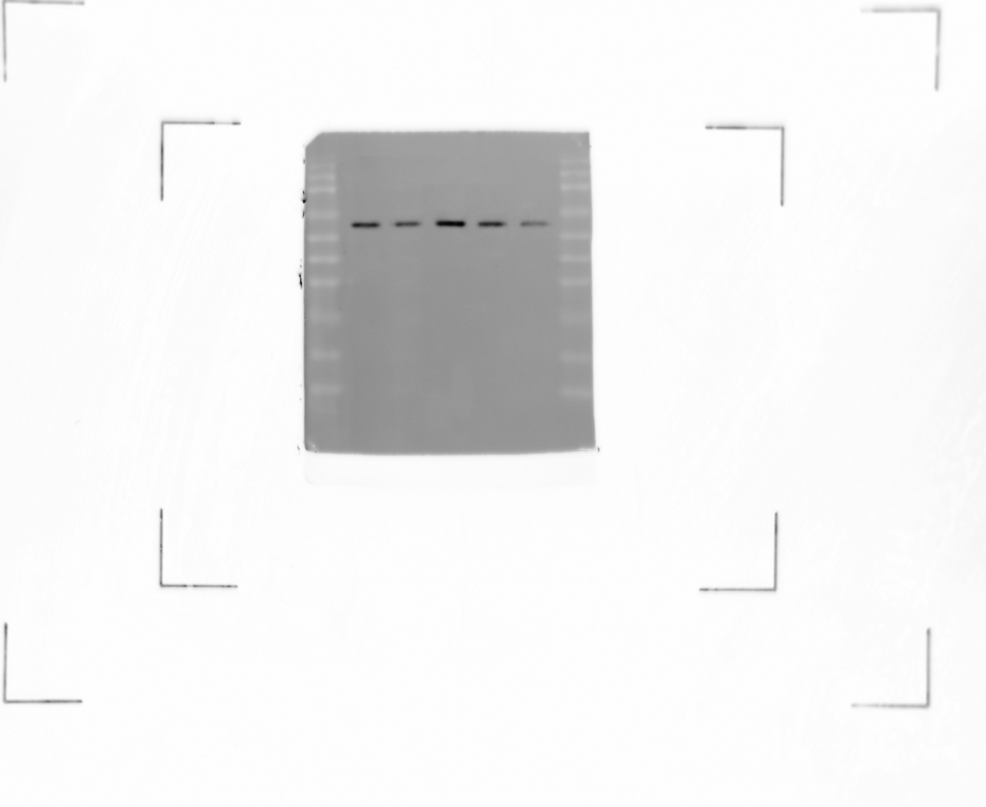

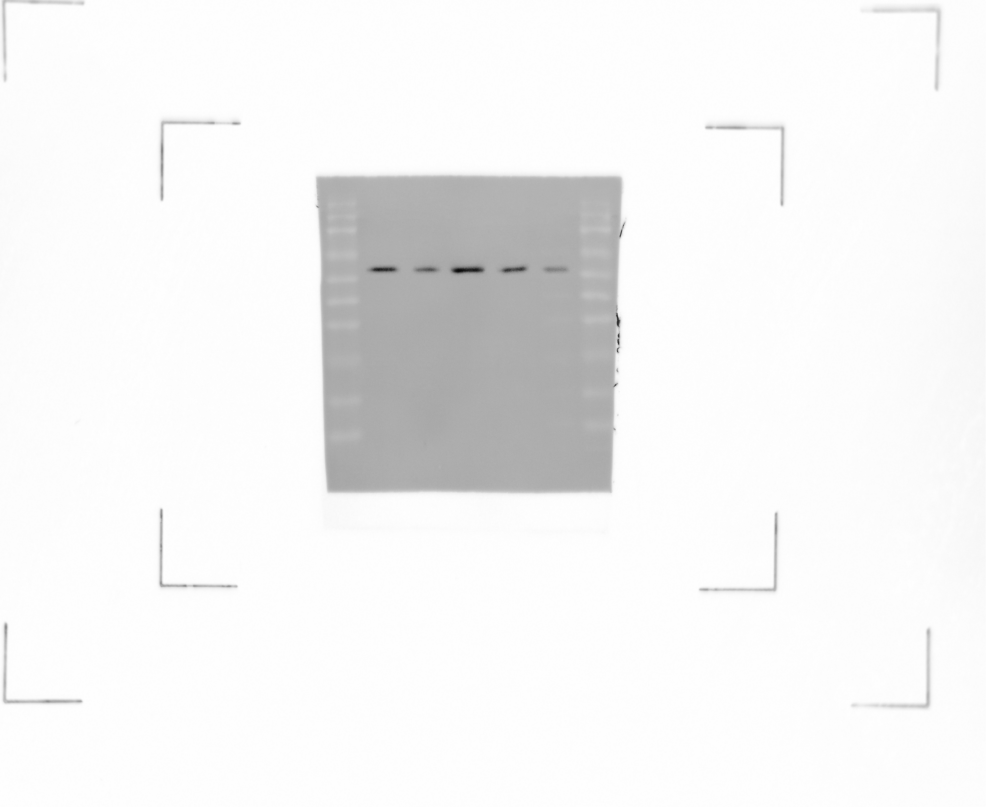


si-NC

Control

si-METTL3#1

pcDNA3.1

pcDNA3.1-METTL3

si-NC

Control

si-METTL3#1

pcDNA3.1

pcDNA3.1-METTL3


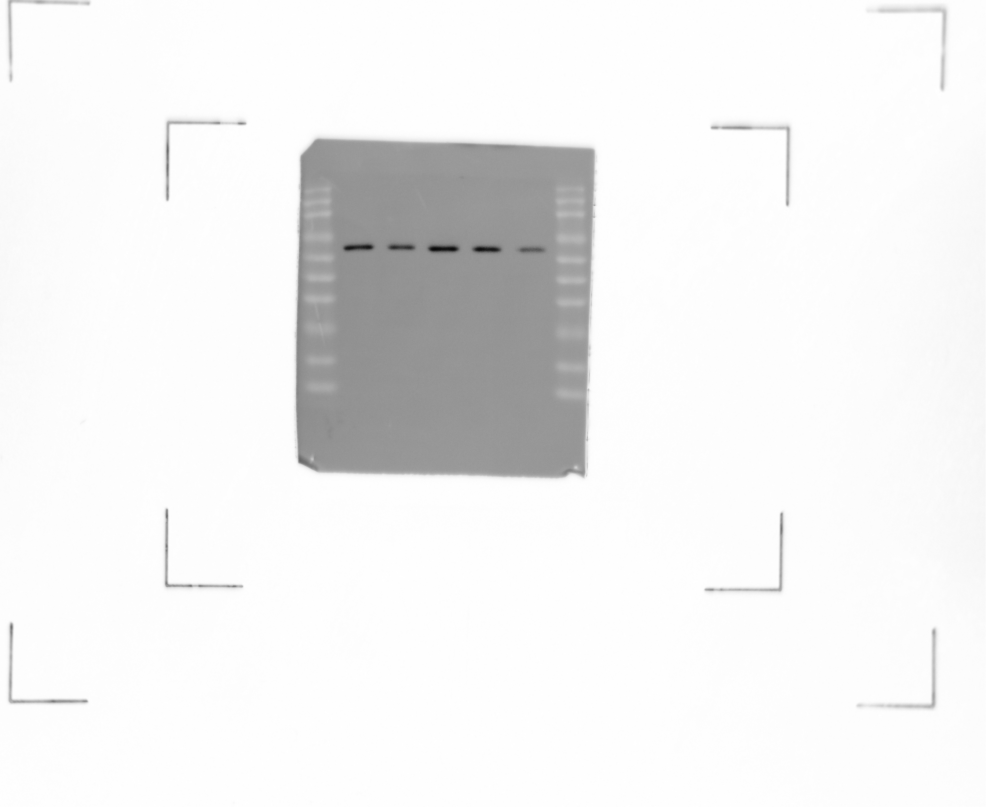


si-NC

Control

si-METTL3#1

pcDNA3.1

pcDNA3.1-METTL3

Cleaved caspase-3


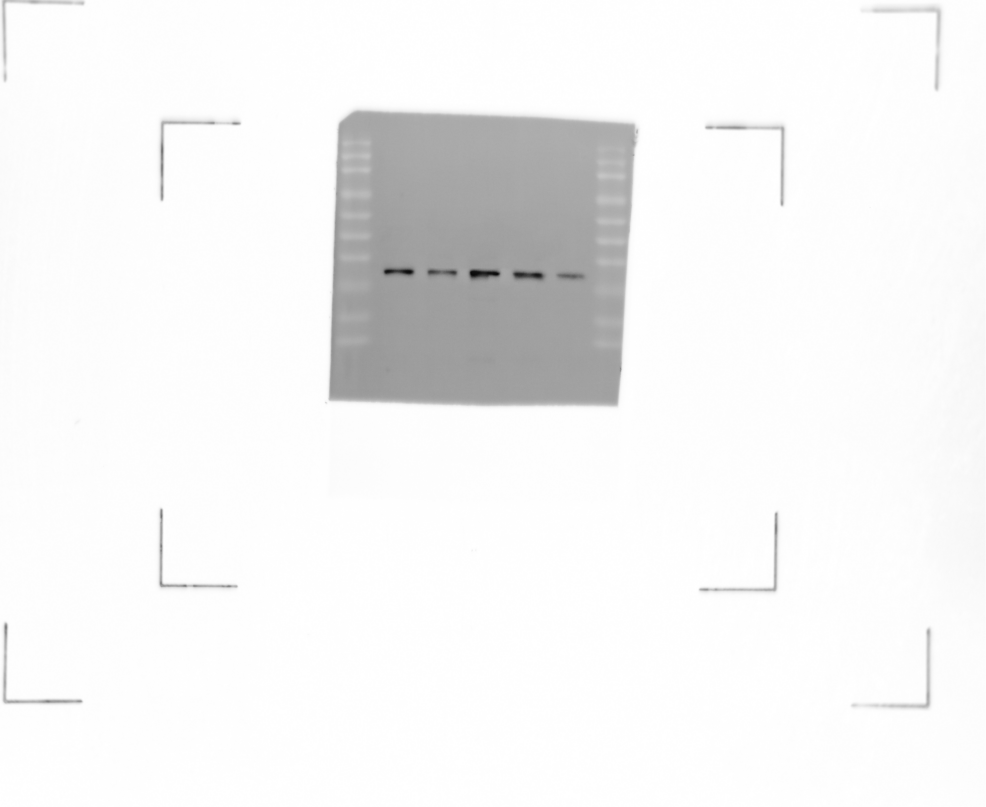

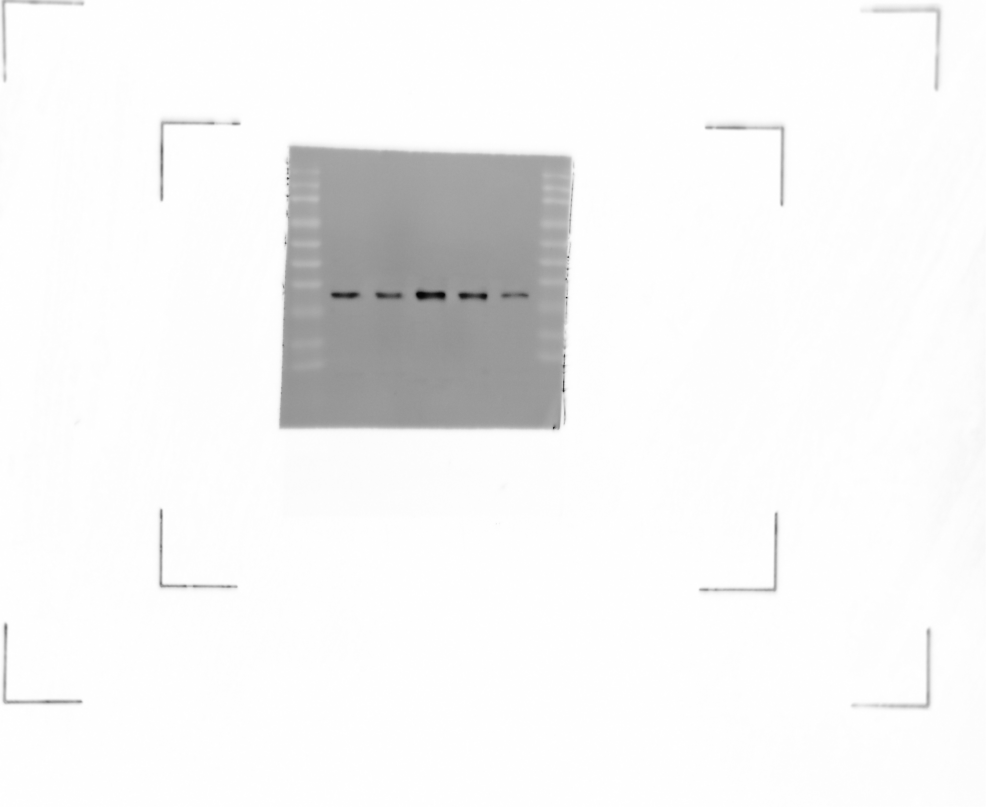


si-NC

Control

si-METTL3#1

pcDNA3.1

pcDNA3.1-METTL3

si-NC

Control

si-METTL3#1

pcDNA3.1

pcDNA3.1-METTL3


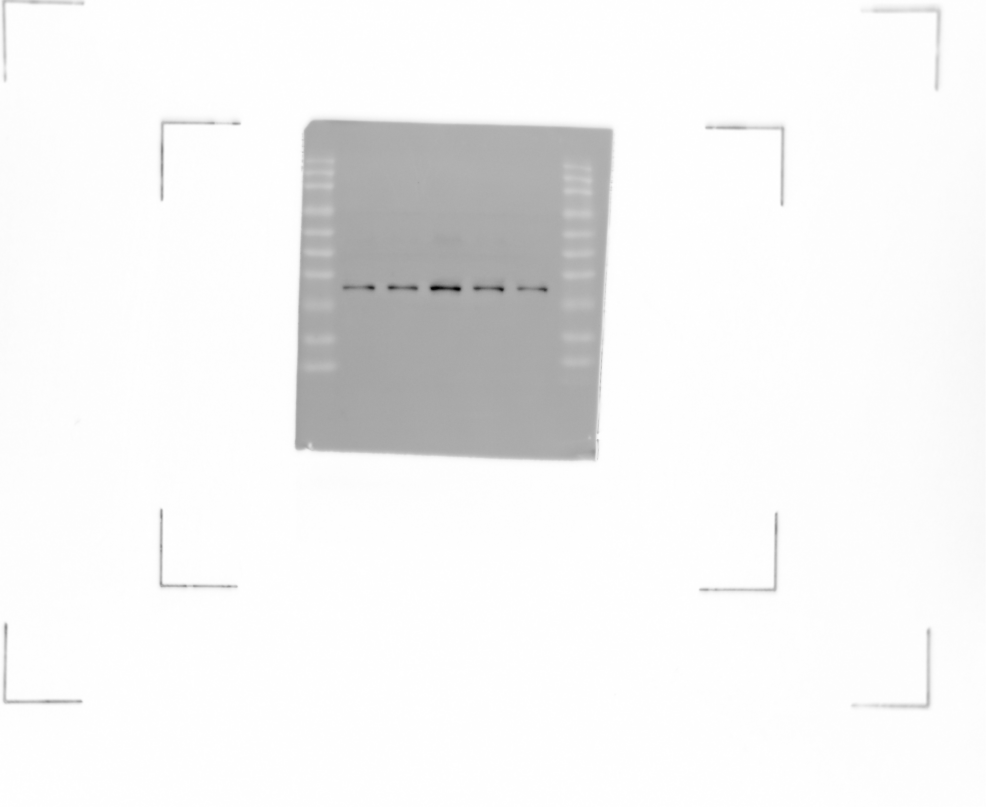


si-NC

Control

si-METTL3#1

pcDNA3.1

pcDNA3.1-METTL3

GAPDH


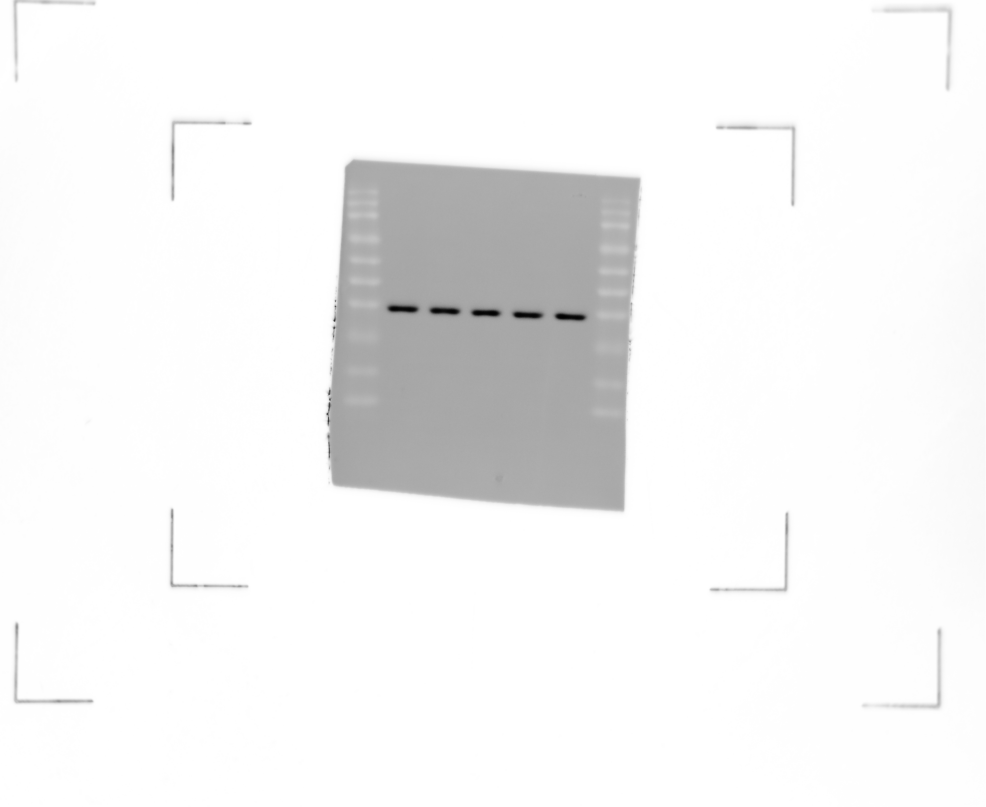

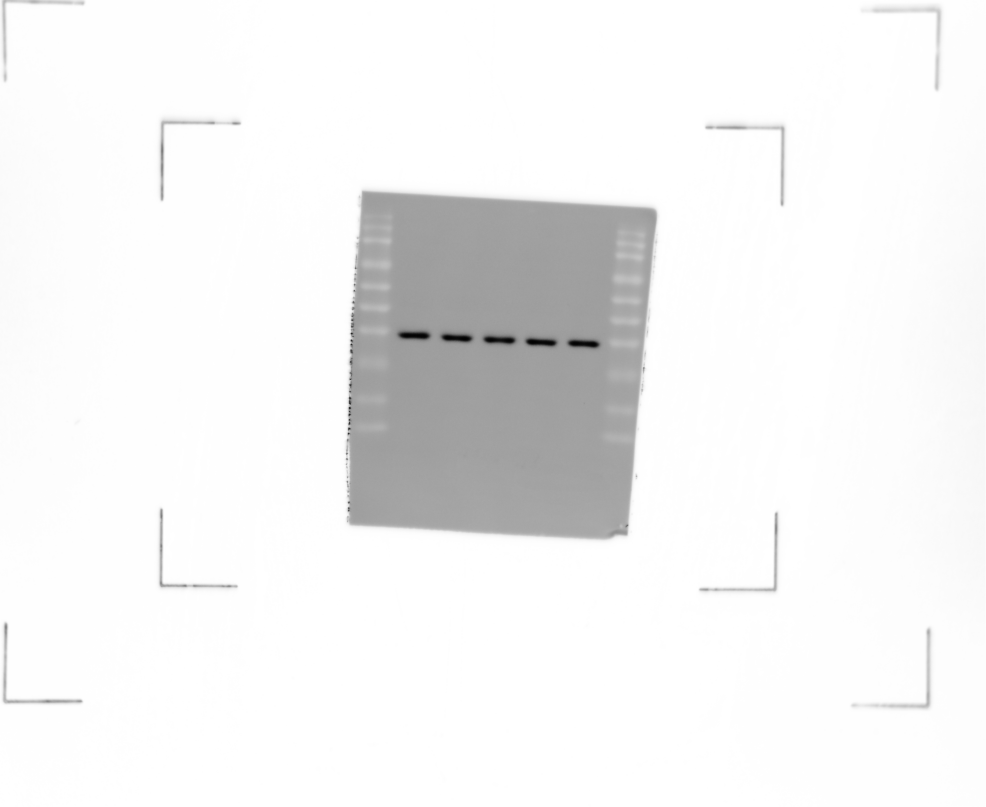


si-NC

Control

si-METTL3#1

pcDNA3.1

pcDNA3.1-METTL3

si-NC

Control

si-METTL3#1

pcDNA3.1

pcDNA3.1-METTL3


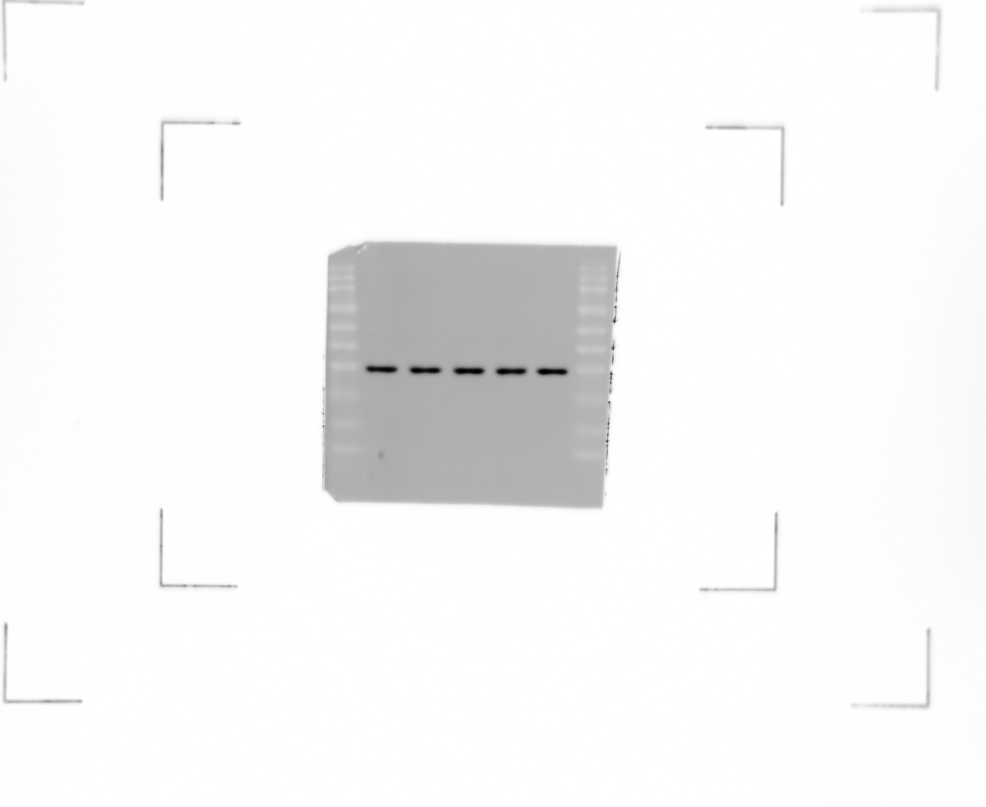


si-NC

Control

si-METTL3#1

pcDNA3.1

pcDNA3.1-METTL3
