# Supplementary material for: Sulforaphene suppressed cell proliferation and promoted apoptosis of COV362 cells in endometrioid ovarian cancer
Source: PeerJ. 2023 Nov 21;11:e16308. doi: 10.7717/peerj.16308 (PMC10668859; doi:10.7717/peerj.16308)
Supplement: Supplemental Information 6 [file peerj-11-16308-s006.docx]

**Original uncropped images of western blots used for the Figure 5d.**

**FAS**


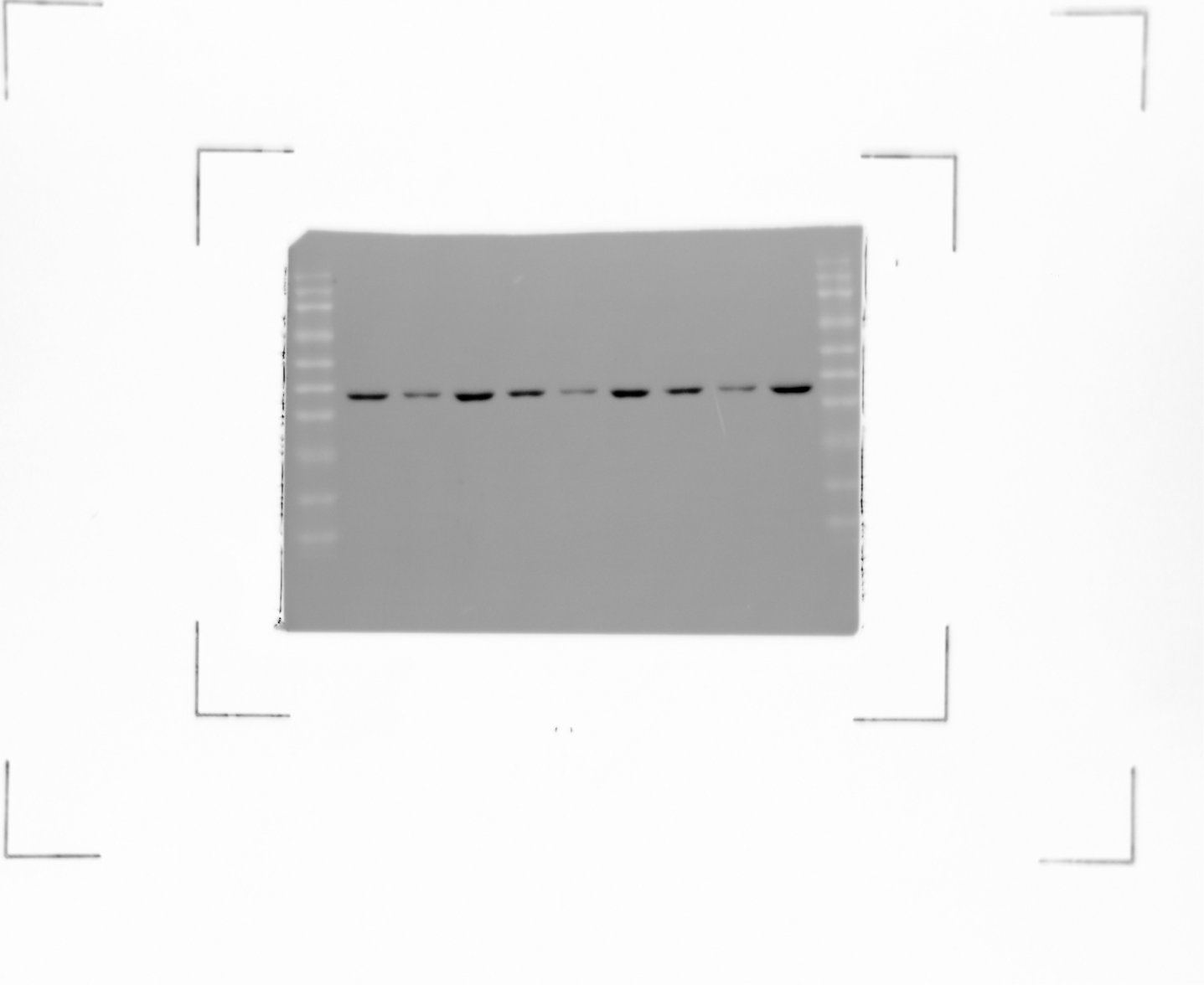


pcDNA3.1

pcDNA3.1-METTL3

pcDNA3.1-METTL3+ Sul (60 μM)

pcDNA3.1

pcDNA3.1-METTL3

pcDNA3.1-METTL3+ Sul (60 μM)

pcDNA3.1

pcDNA3.1-METTL3

pcDNA3.1-METTL3+ Sul (60 μM)

**FADD**


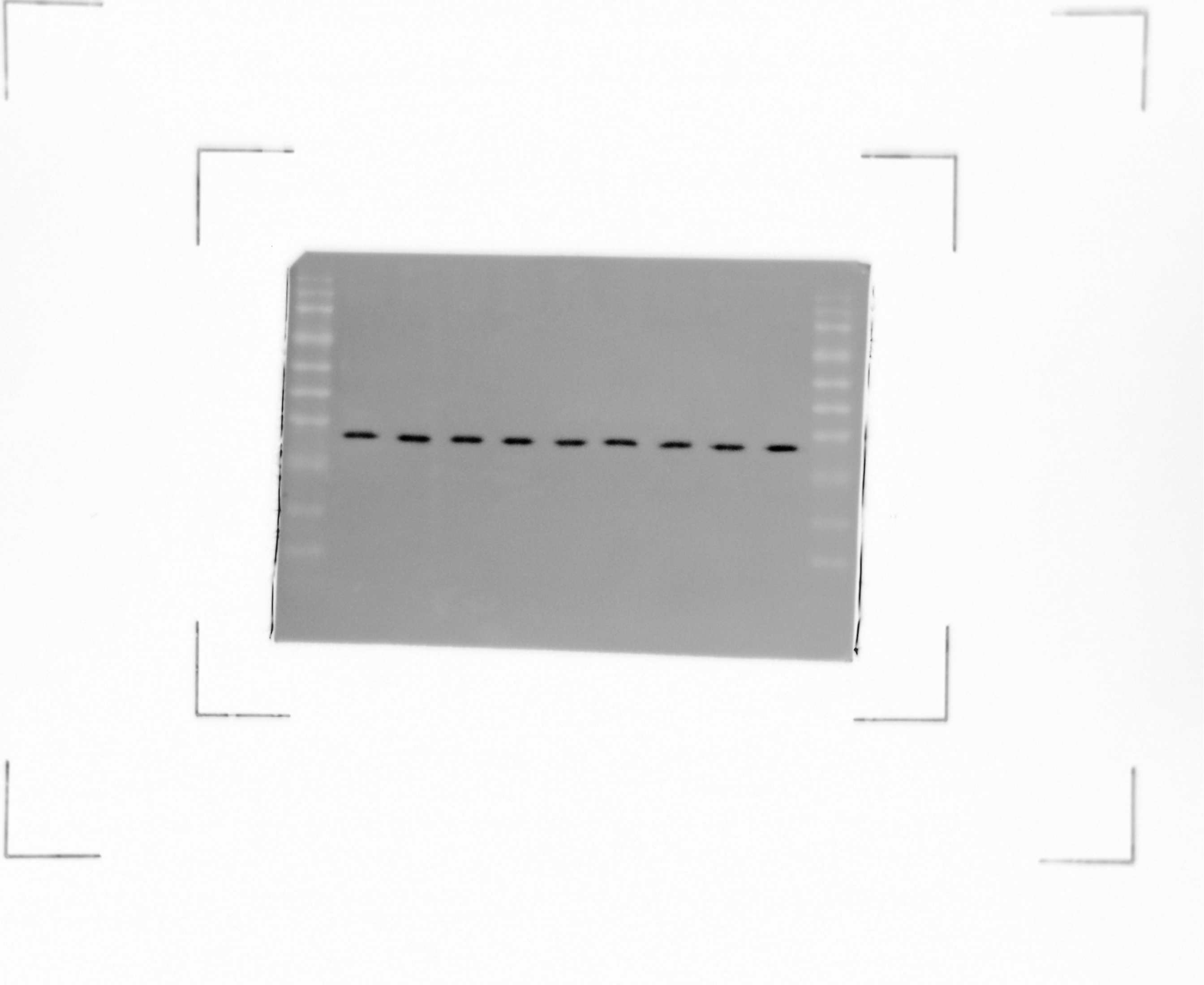


pcDNA3.1

pcDNA3.1-METTL3

pcDNA3.1-METTL3+ Sul (60 μM)

pcDNA3.1

pcDNA3.1-METTL3

pcDNA3.1-METTL3+ Sul (60 μM)

pcDNA3.1

pcDNA3.1-METTL3

pcDNA3.1-METTL3+ Sul (60 μM)

**p-FADD**


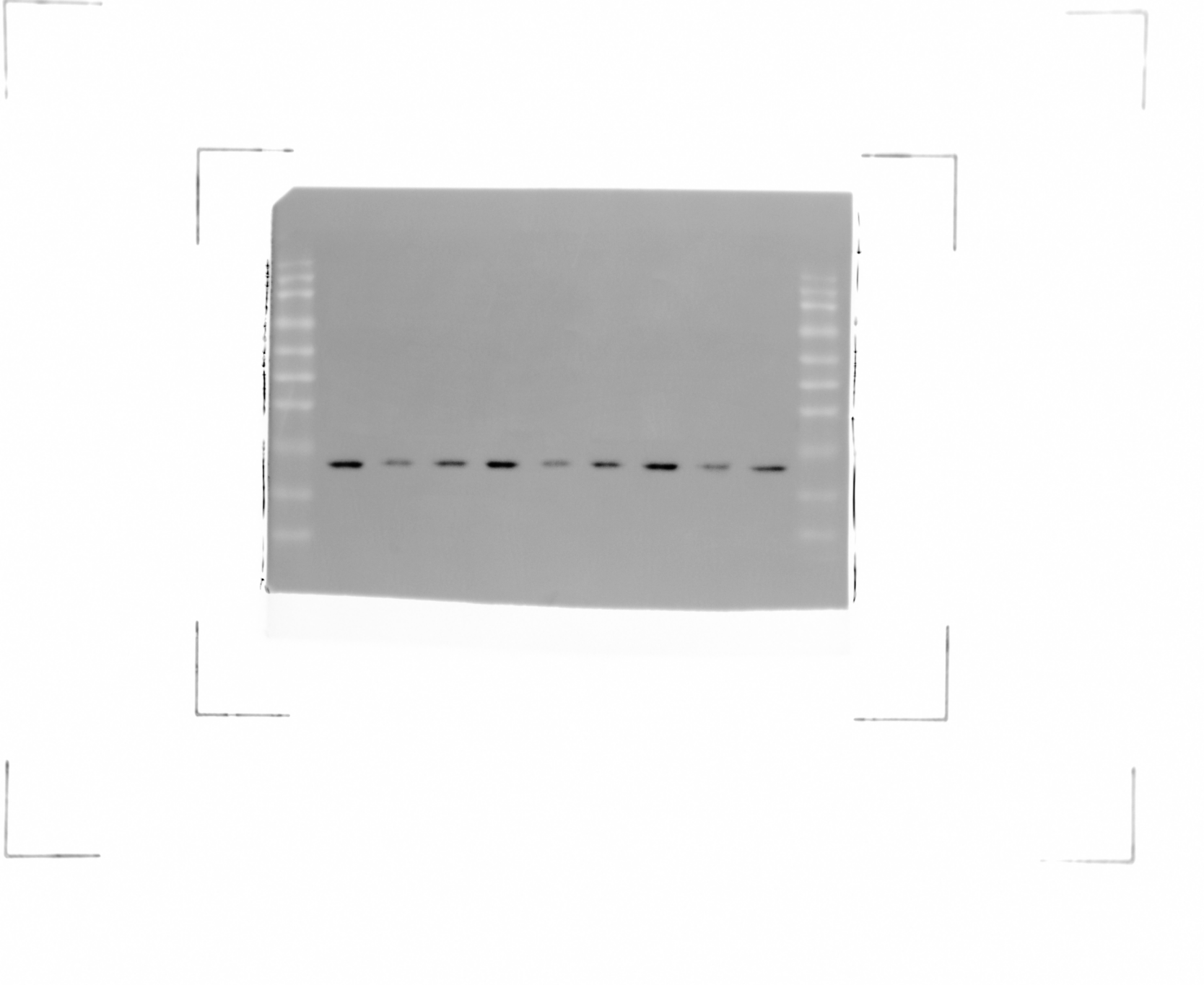


pcDNA3.1

pcDNA3.1-METTL3

pcDNA3.1-METTL3+ Sul (60 μM)

pcDNA3.1

pcDNA3.1-METTL3

pcDNA3.1-METTL3+ Sul (60 μM)

pcDNA3.1

pcDNA3.1-METTL3

pcDNA3.1-METTL3+ Sul (60 μM)

**Bcl-2**


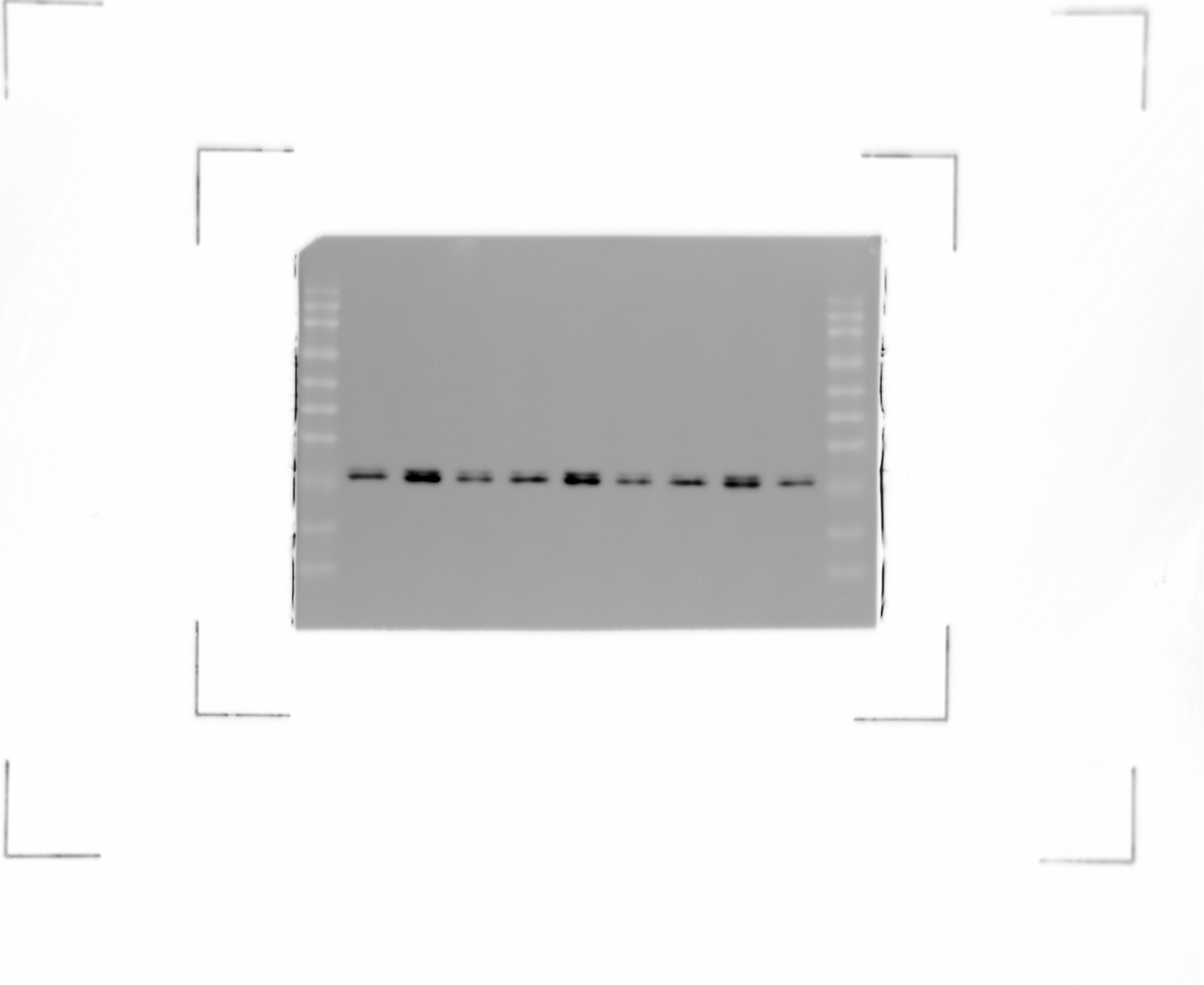


pcDNA3.1

pcDNA3.1-METTL3

pcDNA3.1-METTL3+ Sul (60 μM)

pcDNA3.1

pcDNA3.1-METTL3

pcDNA3.1-METTL3+ Sul (60 μM)

pcDNA3.1

pcDNA3.1-METTL3

pcDNA3.1-METTL3+ Sul (60 μM)

**Bax**


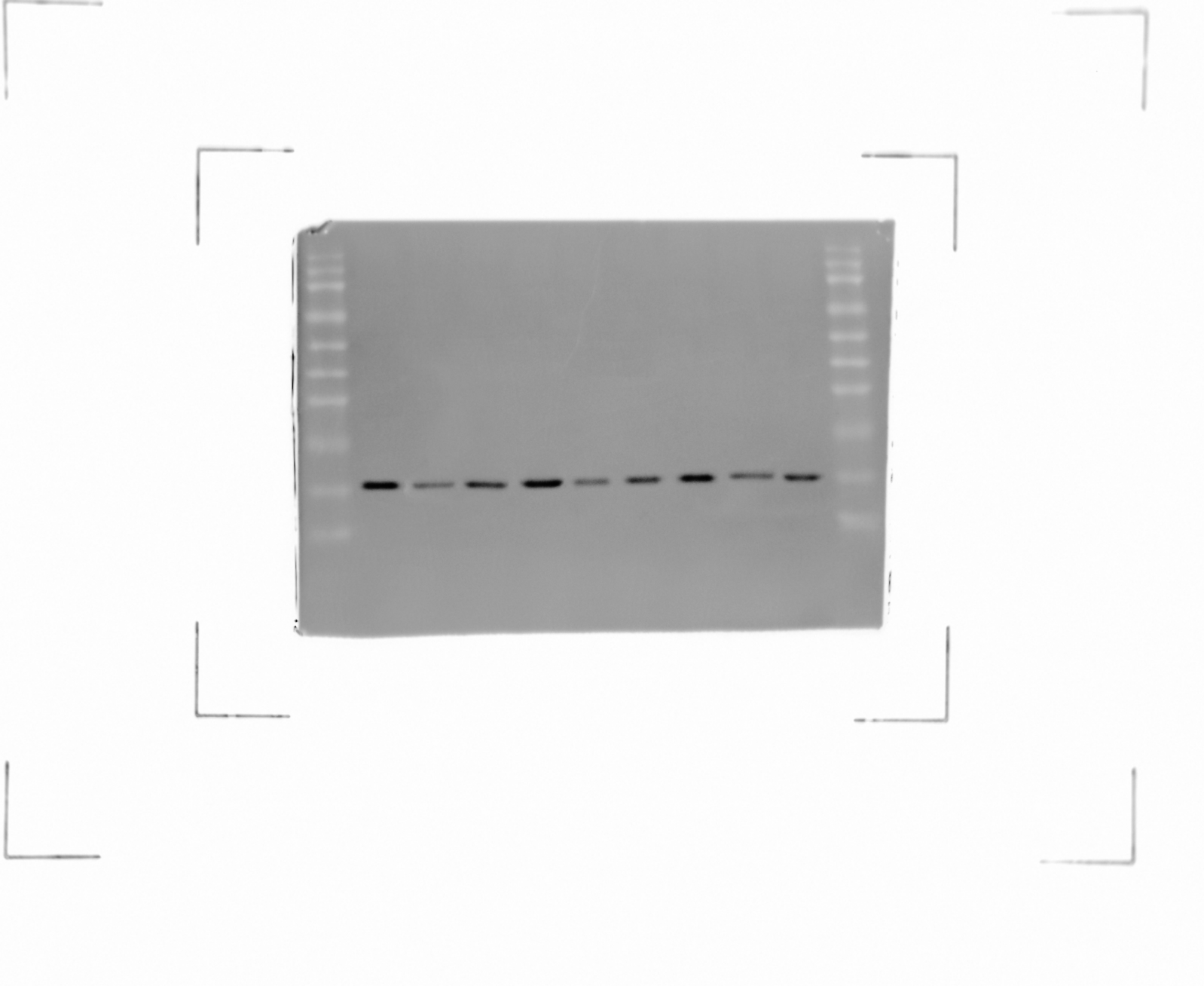


pcDNA3.1

pcDNA3.1-METTL3

pcDNA3.1-METTL3+ Sul (60 μM)

pcDNA3.1

pcDNA3.1-METTL3

pcDNA3.1-METTL3+ Sul (60 μM)

pcDNA3.1

pcDNA3.1-METTL3

pcDNA3.1-METTL3+ Sul (60 μM)

**Cleaved caspase-3**


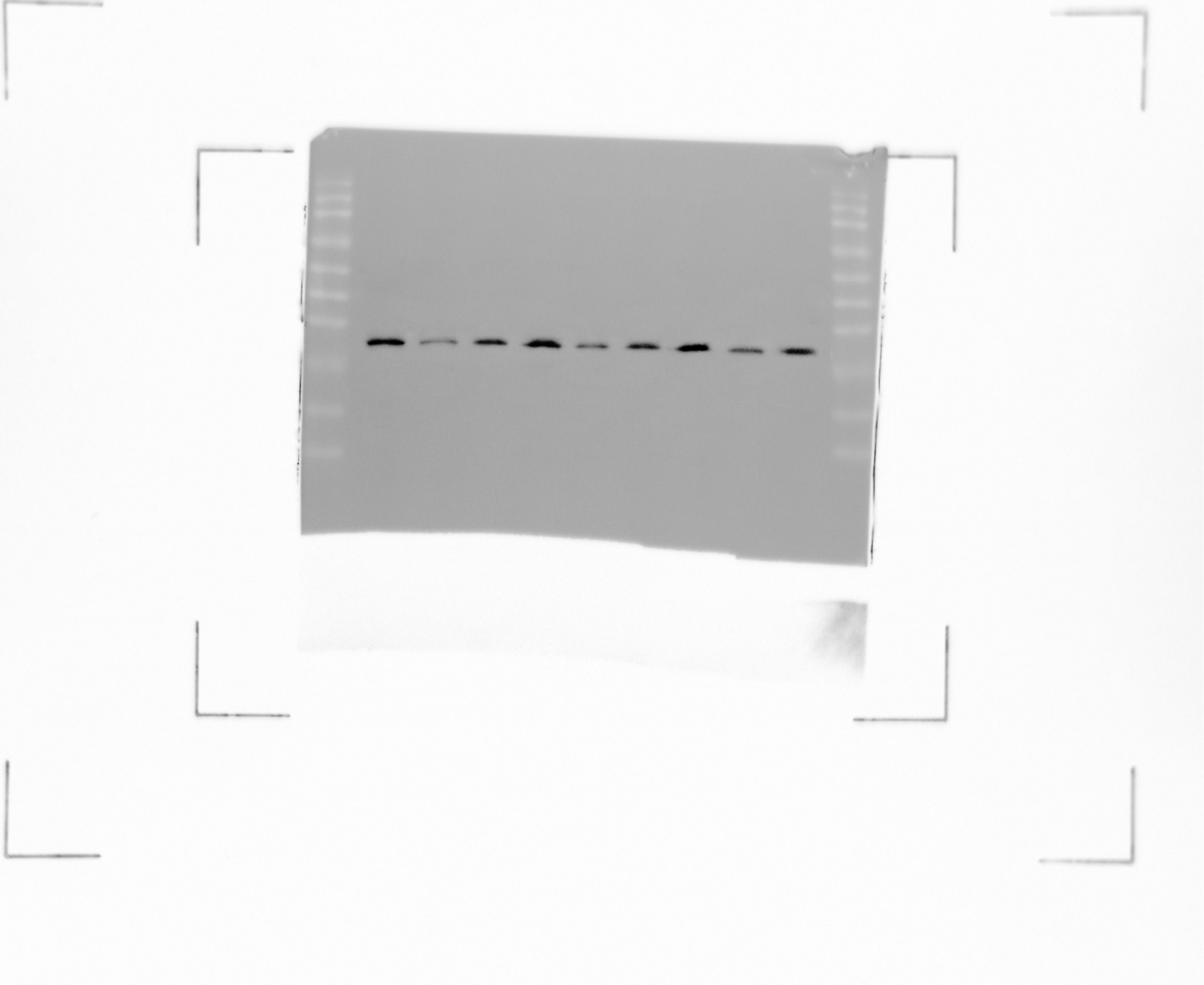


pcDNA3.1

pcDNA3.1-METTL3

pcDNA3.1-METTL3+ Sul (60 μM)

pcDNA3.1

pcDNA3.1-METTL3

pcDNA3.1-METTL3+ Sul (60 μM)

pcDNA3.1

pcDNA3.1-METTL3

pcDNA3.1-METTL3+ Sul (60 μM)

**GAPDH**


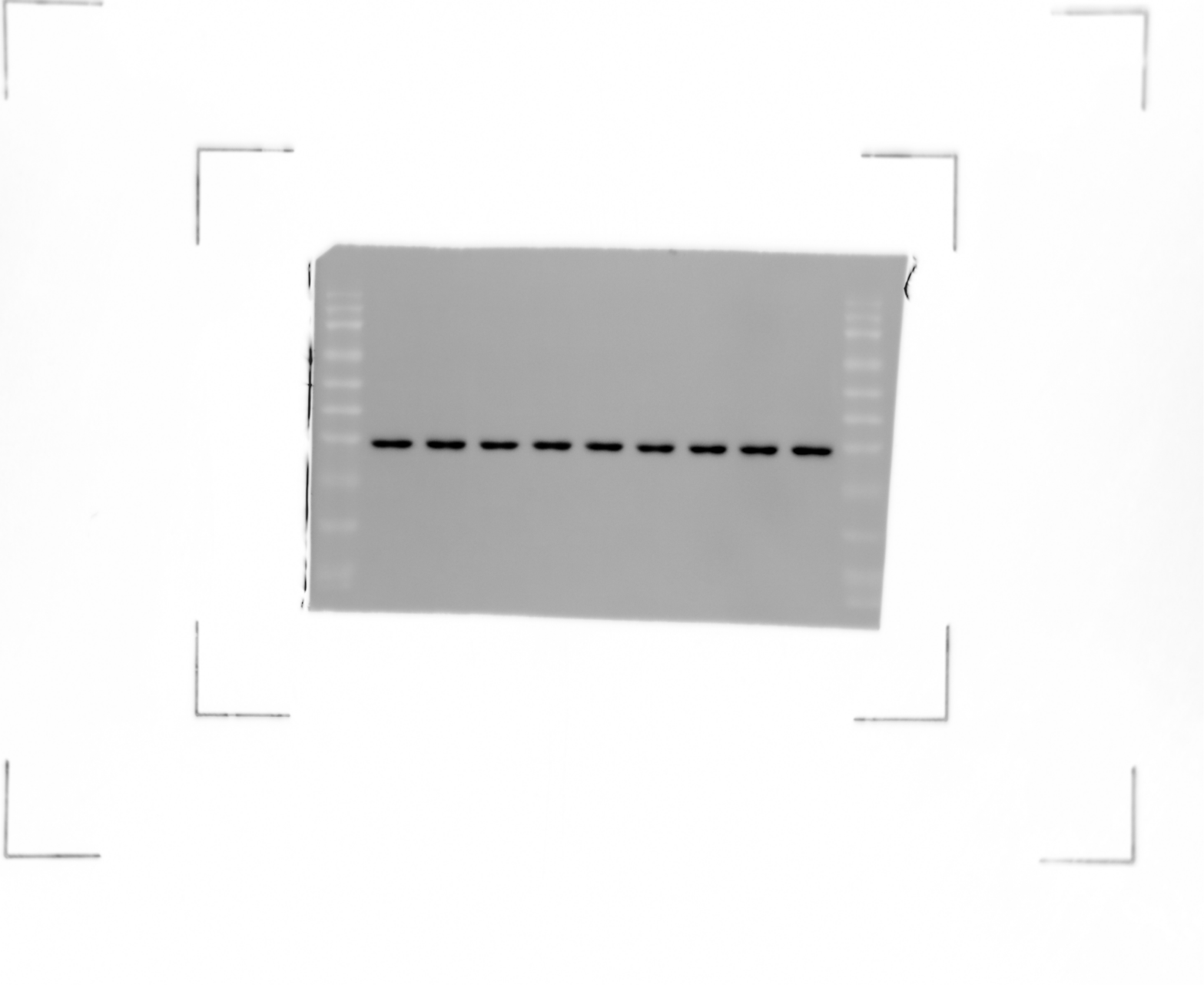


pcDNA3.1

pcDNA3.1-METTL3

pcDNA3.1-METTL3+ Sul (60 μM)

pcDNA3.1

pcDNA3.1-METTL3

pcDNA3.1-METTL3+ Sul (60 μM)

pcDNA3.1

pcDNA3.1-METTL3

pcDNA3.1-METTL3+ Sul (60 μM)
